# Mechanical properties of the brain: Focus on the essential role of Piezo1‐mediated mechanotransduction in the CNS

**DOI:** 10.1002/brb3.3136

**Published:** 2023-06-27

**Authors:** Qingcui Zheng, Hailin Liu, Wen Yu, Yao Dong, Lanqian Zhou, Wenze Deng, Fuzhou Hua

**Affiliations:** ^1^ Department of Anesthesiology the Second Affiliated Hospital of Nanchang University Nanchang Jiangxi P. R. China; ^2^ Key Laboratory of Anesthesiology of Jiangxi Province The Second Affiliated Hospital of Nanchang University Nanchang Jiangxi P. R. China; ^3^ Jiangxi Province Key Laboratory of Molecular Medicine The Second Affiliated Hospital of Nanchang University Nanchang Jiangxi P. R. China

**Keywords:** aging, Alzheimer's disease, brain cells, demyelinating disease, mechanotransduction, Piezo1

## Abstract

**Background:**

The brain is a highly mechanosensitive organ, and changes in the mechanical properties of brain tissue influence many physiological and pathological processes. Piezo type mechanosensitive ion channel component 1 (Piezo1), a protein found in metazoans, is highly expressed in the brain and involved in sensing changes of the mechanical microenvironment. Numerous studies have shown that Piezo1‐mediated mechanotransduction is closely related to glial cell activation and neuronal function. However, the precise role of Piezo1 in the brain requires further elucidation.

**Objective:**

This review first discusses the roles of Piezo1‐mediated mechanotransduction in regulating the functions of a variety of brain cells, and then briefly assesses the impact of Piezo1‐mediated mechanotransduction on the progression of brain dysfunctional disorders.

**Conclusions:**

Mechanical signaling contributes significantly to brain function. Piezo1‐mediated mechanotransduction regulates processes such as neuronal differentiation, cell migration, axon guidance, neural regeneration, and oligodendrocyte axon myelination. Additionally, Piezo1‐mediated mechanotransduction plays significant roles in normal aging and brain injury, as well as the development of various brain diseases, including demyelinating diseases, Alzheimer's disease, and brain tumors. Investigating the pathophysiological mechanisms through which Piezo1‐mediated mechanotransduction affects brain function will give us a novel entry point for the diagnosis and treatment of numerous brain diseases.

## Graphical abstract

Piezo1‐mediated mechanotransduction regulates pathophysiological processes in the brain. Most brain cells, including neurons and glial cells, express mechanosensitive Piezo1 channels. Under normal conditions, Piezo1‐mediated mechanotransduction regulates the direction of neural stem cell differentiation, axonal growth, microglia immunophenotyping, neurogenesis, and myelin regeneration. Under pathological conditions, mechanical signals such as matrix stiffness‐mediated activation of Piezo1 are involved in regulating the development of brain aging, demyelinating diseases, Alzheimer's disease, brain tumors, and traumatic brain injury.

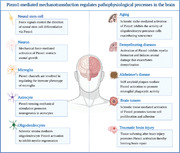

## 1 INTRODUCTION

Brain tissue is one of the softest tissues in the body, with a complex viscoelastic response (Franze et al., 2013; Ivkovic et al., 2022). Although neuroscience is primarily concerned with the regulation of brain function by genetic, biochemical signals, and electrophysiology over the past few decades, emerging evidence confirms that mechanical signals play a key role in regulating brain function and disease (Tyler, 2012). Indeed, accumulating evidence suggests that mechanical properties of the cell microenvironment can regulate physiological processes (Koser et al., 2016) and are also involved in the progression of brain diseases (Procès et al., 2022), whereas external mechanical forces can cause brain dysfunction (Tyler, 2012). For instance, mechanical forces were found to influence normal physiological neuronal network activity (Zhang et al., 2014), axonal extension (Koser et al., 2016), neuron–astrocyte communication (Blumenthal et al., 2014), as well as interactions between neurons and substrates (Franze et al., 2013). In addition, changes in the mechanical properties of the extracellular matrix (ECM) play a crucial part in the progression of brain aging, brain tumors, Alzheimer's disease (AD), and other neurodegenerative diseases (Chen et al., 2018; Pogoda & Janmey, 2018; Segel et al., 2019). Undoubtedly, the study of how mechanical cues are involved in neuropathophysiological processes can provide new research directions for interventions in central nervous system (CNS) diseases.

Mechanical signals induce brain tissue to form a specific stiffness gradient at the cellular scale (Koser et al., 2016), which is determined by ECM molecules and cells embedded in the ECM (Ivkovic et al., 2022; Rutka et al., 1988). Cells are supported structurally by the ECM, which also offers mechanical cues for development and tissue remodeling (Franze et al., 2013; Koser et al., 2016). In the brain, the stiffness of the matrix refers to its hardness or elasticity, which is generally measured by elastic modulus (a measure of tissue resistance to deformation) (Discher et al., 2005; Sarhane et al., 2023). However, roughness refers to the surface structure and topography of the ECM macromolecules and glial support cells (Blumenthal et al., 2014; Brunetti et al., 2010). Indeed, ECM stiffness is an important determinant of neuronal and glial cell growth and function (Rutka et al., 1988). Each differentiated cell has a defined “baseline” level of cellular tension that differentiates in a specific direction in the organizational structure (Wang et al., 1993). Although neurons, astrocytes, and microglia all possess membrane‐bound mechanosensors that respond to different degrees of matrix stiffness, they differ in their mechanotransduction properties based on their cytoskeletal and abundance. Studies have shown that softer gels (∼100–500 Pa) are highly conducive to neurite growth, whereas harder gels (∼1000–10,000 Pa) promote glial growth (Saha et al., 2008). Astrocytes and microglia are small and round on soft (100 Pa) gels while developing into larger, more intensely branched, and highly diffuse cells on stiff (10 or 30 kPa) substrates (Moshayedi et al., 2010; Moshayedi et al., 2014). By contrast, neurons show greater branching and spreading patterns on softer substrates than on stiff substrates (Moshayedi et al., 2010; Pogoda & Janmey, 2018; Teixeira et al., 2009). The migration and phagocytic activity of microglia is also influenced by the extracellular mechanical environment. Harder materials such as amyloid plaques promote microglial phagocytosis and durotaxis (migration toward harder areas) (Blaschke et al., 2020). In addition, the roughness of the environmental substrate has been shown to affect the association between neurons and glial cells in the brain as well as the state of neurons, with excessive roughness leading to neuronal death and uncoupling from astrocytes (Blumenthal et al., 2014). Overall, mechanical signals in the brain regulate cell behavior and morphology, but the extent to which these cellular mechanical dynamics affect brain function, as well as the underlying molecular mechanisms, need to be further elucidated.

Mechanotransduction is a process that converts extracellular mechanical signals into intracellular electrochemical signals (Chalfie, 2009). Mechanotransduction in the CNS is a rapid cellular process, during which cells sense changes in the microenvironment or external mechanical cues through specific mechanosensitive channels (Chalfie, 2009). The Piezo receptor family, which is widely distributed in the CNS, is thought to be crucial for mechanotransduction (Coste et al., 2012). The mechanosensitive ion channel protein Piezo‐type mechanosensitive ion channel component 1 (Piezo1) is one of the most important mechanosignal receptors located on the membrane surface that opens in response to mechanical stimuli (Coste et al., 2012; Coste et al., 2010). Over the past decade, numerous studies confirmed that Piezo1 channels play an essential role in mediating various physiological and pathological processes in the CNS (Harraz et al., 2022; Koser et al., 2016; Murthy et al., 2017; Song et al., 2019). Under homeostasis, Piezo1‐mediated mechanotransduction is indispensable for neurophysiological processes, such as neuronal growth and development (Koser et al., 2016), axon extension (Koser et al., 2016), glial cell migration (Velasco‐Estevez et al., 2020), regulation of glial cell responsiveness (Liu et al., 2021; Velasco‐Estevez et al., 2020), and activation of CNS resident immune cells (Liu et al., 2021). In addition, numerous studies have demonstrated that Piezo1 is involved in neuropathology. First, increased amyloid‐mediated brain roughness in AD patients activates Piezo1 and upregulates Piezo1 protein expression in reactive astrocytes (Velasco‐Estevez et al., 2018). In vitro, Yoda1, a selective activator of Piezo1, was found to directly induce demyelination (Syeda et al., 2015; Velasco‐Estevez et al., 2020). Piezo1 is also involved in the regulation of brain tumor pathology, affecting tumor cell proliferation and migration (Chen et al., 2018; Qu et al., 2020). Given the important role of Piezo1 in neuro(patho)physiology, in this review, we will focus on the basic properties of the mechanosensitive pressure channel Piezo1 and explore its role in the CNS.

## 2 OVERVIEW OF PIEZO1 MECHANOSENSITIVE ION CHANNELS

Back in 2010, the Ardem lab first identified the Piezo family of mechanically gated channel proteins (including Piezo1 and Piezo2), which sense mechanical forces in mammalian Neuro2a cells (Coste et al., 2010). The groundbreaking discovery of Piezo channels enabled us to understand how mechanical forces initiate neuronal impulses that enable the body to perceive and adapt to its surroundings. Piezo1 (also known as Fam38A) is the largest known pore‐type ion channel, which consists of more than 2500 amino acids and weighs 300 kDa (Coste et al., 2010). Mechanical activation of Piezo1 leads to the influx of extracellular Ca^2+^, Na^2+^, and K^+^, which induces the propagation of electrical signals and initiates the intracellular second messenger pathway (Procès et al., 2022). Recent studies used cryoelectron microscopy to analyze the high‐resolution structure and biological function of Piezo1 (Ge et al., 2015; Lin et al., 2019; Saotome et al., 2018; Yang et al., 2022; Zhao et al., 2018). Piezo1 protein possesses a unique 38‐transmembrane helix topology (Coste et al., 2010). Three Piezo1 proteins form homotrimers in the cell membrane, clustered around a central pore (Ge et al., 2015; Zhao et al., 2018). The three proteins spiral outward to form a propeller bladelike structure (Ge et al., 2015; Zhao et al., 2018). The central hole of the Piezo1 trimer is responsible for cation influx, and the three peripheral “propeller blades” are the key area for sensing mechanical forces (Coste et al., 2012; Ge et al., 2015; Guo & MacKinnon, 2017; Zhao et al., 2018). This structure is the basis for the mechanisms through which Piezo1 senses mechanical forces and translates them into intracellular electrochemical signals.

**FIGURE 1:**
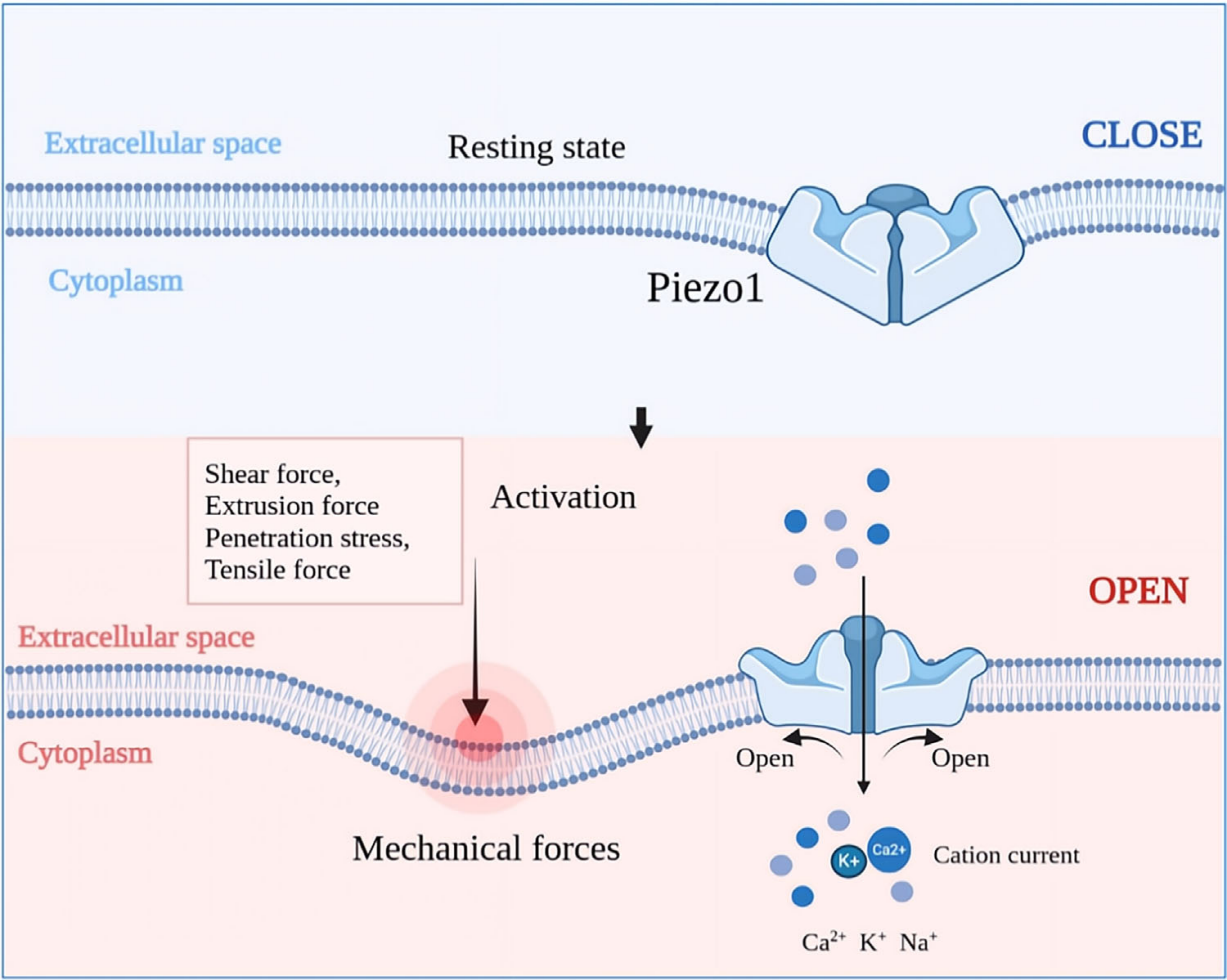
**Schematic diagram of the state of Piezo1 channels at rest and activation**. The Piezo1 channel protein is a trimeric structure embedded in the lipid membrane. In the resting state, the propeller bends outward to close the central ion channel. Forces from the lipid membrane drive the transition of Piezo1 from the curved state to a fully flattened state, in with the central pore is opened to allow the influx of cations.

Piezo1 channels have three interchangeable states: closed, open, and inactivated. At rest, Piezo1 is embedded in the cell membrane and the propeller bends outward, allowing the central ion channel to close, whereas transient mechanical signals stimulate Piezo1 to open reversibly and finally inactivate completely within 100 ms (Figure 1) (Romero et al., 2019). Structural analysis system of membrane stress demonstrated that the three propeller blades of Piezo1 open the central pore when the membrane is stressed in the plane, thus inducing a mechanically activated cationic current (Yang et al., 2022). Overall, Piezo1 is activated by sensing the mechanical indentation and tension of the cell membrane, translating the cell‐side membrane tension into free energy‐dependent molecular conformational changes. In addition to membrane tension, the composition of the membrane also affects the state of Piezo1, as membranes rich in saturated fatty acids inhibit Piezo1 activation, whereas those rich in polyunsaturated fatty acids induce prolonged inactivation of Piezo1 channels (Romero et al., 2019). In addition, it has been demonstrated that the response of Piezo1 to mechanical pressure is dependent on an intact cytoskeleton (Gottlieb et al., 2012; Maneshi et al., 2018). Independent of membrane mechanics, recent studies have shown that large extracellular H^+^ concentrations enhance the occupancy of Piezo1 channels in the closed or inactive state, indicating that low‐pH environments (pH < 6.5) also induce Piezo1 inactivation (Bae et al., 2015).

**TABLE 1:** The function of Piezo1 in different brain cells.

Target cells/animals	Study	Interference factors	Variation of Piezo1	Functions	Related mechanisms	Piezo1 inhibition/Ko
NSCs	Blumenthal et al. (2014)	Topography (Rq = 32)	Activating	Differentiation to neurons	Intracellular Ca^2+^ oscillations	Inhibition of differentiation toward neurons
hNSPCs	Pathak et al. (2014)	Stiff matrix (>5 kPa)	Activating	Differentiation to neurons	Promotes Ca^2+^ influx and nuclear localization of Yap	Differentiation to astrocytes
NSCs	Nourse et al. (2022)	Piezo1Ko	Absence	Inhibits proliferation and differentiation	Inhibits the synthesis of cholesterol	/
NSCs	Li et al. (2022)	MNBs/LIPUS	Activating	Promotes differentiation and maturation	Promotes BMPs/Smad phosphorylation	/
PC12	Blumenthal et al. (2014)	Topography (Rq > 60)	Activating	Neuronal death	/	Cell survival
DRG neurons	Li et al. (2021)	Lower substrate stiffness (0.3–1 kPa)	Activating	Inhibits axonal growth	Activation of the Atr‐Chek1 pathway	Showing more total neurite length
*Xenopus laevis* RGC	Koser et al. (2016)	Stiff substrates	Upregulate	Promotes axonal growth	/	Aberrant axonal growth and pathfinding errors
DA sensory neuron/rat hippocampal neurons	Song et al. (2019)	Axonal injury/Piezo1 overexpression/Yoda1	Upregulate	Inhibition of axonal regeneration	Promote the CamKII‐Nos‐PKG signaling	Promotes axonal regeneration
*Piezo1* KO mouse model	Nourse et al. (2022)	Piezo1 KO	Absence	Aberrant neuroepithelial development	Inhibits the synthesis of cholesterol	Brain Aβ load and cognitive impairment
Microglia/5×FAD mouse	Hu et al. (2023)	Stiff amyloid plaques and Yoda1	Upregulate	Promotes microglia aggregation and phagocytosis and cognition	/	Exacerbates AD‐like pathologies
*iMGL*	Jäntti et al. (2022)	Yoda1	Upregulate	Promotes microglia phagocytosis, migration and chemotaxis	Intracellular Ca^2+^ oscillations	Promotes microglia death
5xFAD	Jäntti et al. (2022)	Yoda1	Upregulate	Inhibits Aβ deposition	Regulates Ca^2+^‐related signals	/
BV2/mouse primary microglia	Zhu et al. (2023)	Yoda1/stiff substrates	Upregulate	Inhibits microglia migration and promotes immune response	Regulates Ca^2+^‐related signals	Inhibits of the immune response promotes microglia migration
BV2/ mouse	Liu et al. (2021)	Hyperglycemia/hypertonic	Upregulate	Inhibits microglia function and inflammatory factors release	Regulates Ca^2+^‐related mTOR, JNK1 pathway	Improves microglia function
BV2 and primary microglia	Malko et al. (2023)	Yoda1	Upregulate	Downregulates the pro‐inflammatory function of microglial	Inhibits the NF‐κB inflammatory signaling pathway	Removed the effect of Yoda1
Astrocyte Piezo1 KO mice	Chi et al. (2022)	Piezo1 KO	Absence	Cognitive deficits, impaired neurogenesis	Inhibits Ca^2+^‐dependent ATP release	/
Astrocytes/mouse	Velasco‐Estevez et al. (2020)	LPS + Yoda1	Upregulate	Inhibition of LPS‐induced cytokine release	Promotes Ca^2+^ oscillations	/
Neonatal OPCs/Rat	Segel et al. (2019)	Stiff substrates/the aged niche	Activating	Proliferated and differentiated poorly	/	Promotes proliferated and differentiated
Human MO3.13 OP	Velasco‐Estevez et al. (2022)	Yoda1	Activating	Inhibits oligodendrocyte proliferation and migration	/	Promotes oligodendrocyte proliferation and migration

Abbreviations: AD, Alzheimer's disease; ATP, Adenosine triphosphate; DRG, dorsal root ganglion; hNSPCs, human neural stem/progenitor cells; LPS, lipopolysaccharide; NSCs, neural stem cells; OPCs, oligodendrocyte progenitor cells; RGCs, retinal ganglion cells.

Piezo1 channel sensitivity is regulated by resting membrane tension and is correlated with the cell type and cytoskeleton (Lewis & Grandl, 2015; Saotome et al., 2018; Syeda et al., 2016; Yang et al., 2022). In addition to localization to the surface of the cell membrane, Piezo1 is also distributed in the endoplasmic reticulum (ER) and cytoplasmic compartments, as well as in the nuclear envelope (Coste et al., 2012; Coste et al., 2010; Gudipaty et al., 2017; Mchugh et al., 2010). This ensures that cells are able to sense a variety of “inside‐out” and “outside‐in” mechanical forces, including radial pressure, membrane tension, shear stress, matrix stiffness and topography, ultrasound, and osmotic pressure (Liu et al., 2022). Notably, the distribution, localization, and abundance of Piezo1 in cells are altered by mechanical forces, contributing to the cellular response to mechanical forces (Gudipaty et al., 2017).

## 3 THE ROLE OF PIEZO1‐MEDIATED MECHANOTRANSDUCTION IN PHYSIOLOGICAL PROCESSES IN THE CNS

The execution of normal brain functions depends on a stable mechanical environment (Tyler, 2012). Mechanical cues within the brain and external stimuli act synergistically to regulate cell behavior (including cell adhesion, spreading, proliferation, migration, and differentiation) at different scales (Sarhane et al., 2023). During this process, mechanoreceptors integrate mechanical signals into biochemical signals and genomic pathways leading to observable effects on brain cell fate (Procès et al., 2022). There is increasing evidence that the regulation of cell behavior by mechanical forces is partly dependent on the Piezo1 channel protein (Blumenthal et al., 2014; Chen et al., 2018; Koser et al., 2016; Pathak et al., 2014; Segel et al., 2019; Zhu et al., 2023). In the next section, we will explore the different roles of Piezo1 channels in different brain cells.

### 3.1 Role of Piezo1 in neural stem cells

**FIGURE 2:**
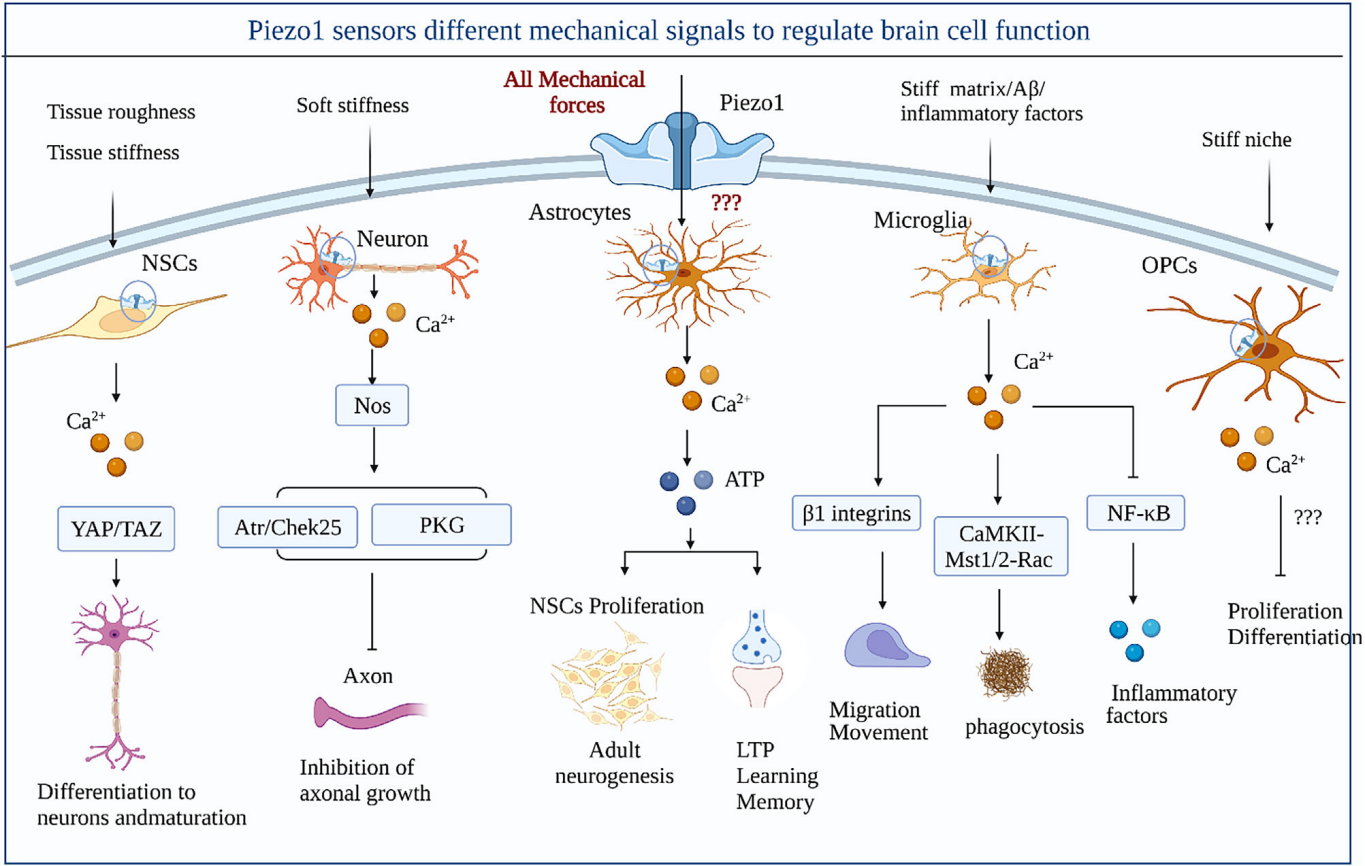
**The different roles of Piezo1 channels in different types of brain cells**. The Piezo1 channel in neural stem cells (NSCs) senses changes in the roughness and stiffness of the substrates. At a roughness corresponding to healthy astrocytes, NSCs tend to differentiate into neurons. Under mechanical stimulation (e.g., ultrasound, matrix stiffness, and roughness), Piezo1 is activated and promotes the differentiation and maturation of NSCs by facilitating the nuclear localization of yes‐associated protein (YAP). In the neuronal axon, a softened matrix induces Piezo1 activation, which promotes Ca^2+^ release and NOS activation, after which NO enters the nucleus as a second messenger to activate the Atr/Chek25 and cGMP‐dependent protein kinase G (PKG) pathways, thereby inhibiting axonal regeneration. Among the physiological functions of astrocytes, Piezo1 channels mediate the release of adenosine triphosphat (ATP) and Ca^2+^ to promote neurogenesis, which improves hippocampal learning and memory. However, the source of mechanical stimulation of activated astrocytes Piezo1 during brain development is unclear. Piezo1 in microglia is activated by mechanical stimuli such as stiffened amyloid plaques or extracellular matrix (ECM), but also in response to pro‐inflammatory factors such as amyloid β (Aβ) or lipopolysaccharide (LPS). Piezo1‐mediated Ca^2+^ influx maintains cytoskeletal plasticity and promotes microglial migration. The CaMKII‐Mst1/2‐Rac axis is activated by Piezo1‐mediated Ca^2+^ influx, which promotes microglial phagocytosis. Piezo1‐mediated Ca^2+^ influx suppresses the microglial cellular immune response by inhibiting the NF‐κB pathway. In addition, the Piezo1 channel mediates the differentiation and regeneration of oligodendrocyte progenitor cells (OPCs).

The morphology of the brain support cells (e.g., astrocytes) and the structure and composition of the ECM provide physical cues for the growth and differentiation of neural stem cells (NSCs) (Blumenthal et al., 2014; Gu et al., 2009; Weissmüller et al., 2000). Notably, the different origins of the different cell types (e.g., human vs. mouse or fetal vs. adult) affect the mechanosensitive differentiation. Human neural stem/progenitor cells (hNSPCs) tend to differentiate into neuronal cells on harder (750 kPa) substrates, whereas in rat NSCs, soft substrates (<1 kPa) support more neurogenesis than harder substrates (>5 kPa) (Pathak et al., 2014; Saha et al., 2008). NSCs convert extracellular mechanical signals into intracellular pathways that affect NSC differentiation through the Piezo1 channel protein (Blumenthal et al., 2014; Pathak et al., 2014). Pathak et al. (2014) showed that myosin II‐dependent traction forces mediate the activation of Piezo1 channels in hNSPCs on stiff substrates. Activation of Piezo1 channels induces transient Ca^2+^ currents and promotes the nuclear localization of the transcriptional coactivators yes‐associated protein (YAP)/PDZ‐binding motif (TAZ) to facilitate the differentiation of hNSPCs into neurons (Pathak et al., 2014). The YAP/TAZ pathway is a part of the Hippo pathway, and their nuclear–cytoplasmic localization affects cell proliferation, differentiation, and survival (Figure 2) (Dupont et al., 2011). Therefore, the Piezo1/YAP/TAZ pathway may be one of the hubs linking extracellular mechanical signaling and cell function.

Consistent with substrate stiffness, the roughness also affects the differentiation of NSCs, and this process is largely dependent on the Piezo1 channel (Blumenthal et al., 2014). Studies have shown enhanced differentiation of telencephalic NSCs into neurons in an environment with a roughness corresponding to healthy astrocytes (Blumenthal et al., 2014). NSCs can differentiate into neurons even in chemical environments that facilitate differentiation into astrocytes, such as exposure to a cholinergic neurotrophic factor, and blocking Piezo1 with GsMTx4 inhibits the ability of neurons to perceive the roughness of astrocyte tips (Blumenthal et al., 2014). Thus, Piezo1‐mediated mechanical cues, but not chemical signals, contribute significantly to the direction of differentiation of NSCs. Recently, Li et al. (2022) investigated the effect of magnetic nanobubbles (MNBs) on the stiffness and differentiation of NSCs. MNBs are a nanomaterial that enters the cells and causes an increase in cellular stiffness to open the Piezo1 mechanosensor in NSCs (Li et al., 2022). Piezo1 activation mediates Ca^2+^ inflow to induce the phosphorylation of bone morphogenetic proteins (BMPs)/Smad, thereby promoting directional differentiation of NSCs (Li et al., 2022). The mechanical force mediated by low‐intensity pulse ultrasound further activates the pathway to accelerate NSC differentiation and synaptic maturity (Li et al., 2022). BMP signaling has a diverse set of functions and influences NSC fate, differentiation, and maturation at nearly every stage of neural development (Bond et al., 2012). The discovery of the Piezo1/BMPs/Smad pathway further supports the key role of Piazo1 in regulating neural development.

A recent study described the mechanism through which Piezo1 regulates neurogenesis in NSCs (Nourse et al., 2022). The authors showed that the knockdown of Piezo1 inhibited the cholesterol biosynthesis pathway, thereby suppressing NSC proliferation and differentiation (Nourse et al., 2022). This suggests an important role for Piezo1 in the maintenance of neural development. The key point of NSC transplantation for the treatment of neurological disorders is to direct the differentiation of transplanted cells. For example, in a photochemically induced mouse model of stroke, transplantation of MNB‐NSCs promoted the maturation of peri‐infarct neurons and vascularization (Li et al., 2022). Pharmacological agents that regulate Piezo1 activity may help direct the differentiation of transplanted NSCs into the desired lineage.

### 3.2 Piezo1‐mediated mechanotransduction regulates axon growth

Mechanical signals are critical for neuronal axon migration and growth in the developing brain, whereas substrate rigidity affects the direction of axon migration and their growth rate (Koch et al., 2012; Koser et al., 2016; Lo et al., 2000). Through Piezo1, neuronal axons perceive external mechanical cues that guide axonal growth (Li et al., 2021). It was shown that the dorsal root ganglion (DRG) neuropil of adult mice has maximum extension and traction at a matrix stiffness of 1 kPa (Koch et al., 2012). Piezo1 was activated on DRG neurons at a substrate stiffness of 0.3–1 kPa, and within this range, a lower substrate stiffness was more conducive to Piezo1 activation (Li et al., 2021). However, neuronal axon growth and migration capacity increase with substrate stiffness (Koser et al., 2016; Li et al., 2021; Procès et al., 2022). This suggests that Piezo1 activation is negatively correlated with axon growth and migration. Mechanistically, Li et al. (2021) showed that Piezo1 mediates nitric oxide synthase (NOS) activation and induces the Atr/Chek25 pathway, thereby inhibiting axonal regeneration. This is consistent with a report by Song et al. (2019), who found that Piezo1 activation inhibited regeneration after axonal injury in the *Drosophila* dendritic arborization sensory neuron injury model, whereas knockdown of Piezo1 promoted axonal regeneration. Further research showed that nerve injury‐mediated Piezo1 activation inhibits axonal regeneration through activation of the Ca^2+^/calmodulin‐dependent protein kinases (CamKII)/NOS/cGMP‐dependent protein kinase G (PKG) pathway (Figure 2) (Song et al., 2019). Both studies highlighted the negative effect of the Piezo1‐mediated release of NO on axonal growth. The modulus of elasticity (a measure of tissue resistance to deformation) of healthy rat cortical tissue ranges from 0.05 to 5 kPa and is even lower after brain injury (Moeendarbary et al., 2017). This suggests that injury‐induced brain softening allows the elastic modulus to fall within the range of Piezo1 activation thereby inhibiting neuronal axon growth (Song et al., 2019). In summary, neuronal perception of substrate rigidity is dependent on the mechanosensitive ion channel Piezo1, and activation of Piezo1 inhibits axonal growth.

However, Koser et al. (2016) discovered that Piezo1 is distributed along axons and growth cones of *Xenopus* retinal ganglion cells (RGCs) and is upregulated in the “stiff” mechanical environment, favoring axonal growth. However, knockdown or inhibition of Piezo1 led to aberrant axonal growth and pathfinding errors (Koser et al., 2016). However, *Drosophila* and mouse sensory neuron‐specific conditional Piezo knockouts show no significant defects in axon development or guidance (Song et al., 2019). This may be attributed to interspecies differences, which have different cytoskeletal organizations, with different distributions and densities of mechanosensitive channels, leading to differences in their mechanical sensitivity (Lo et al., 2000).

Although RGC axons grow faster, straighter, and more parallel on harder substrates, the axons tend to grow in the direction of softer substrates (Koser et al., 2016). Lower stiffness, which promotes slower exploratory growth and axon splaying, appears to be beneficial in regions where axons must search for their targets and form synapses (Koser et al., 2016). It is worth noting that the local stiffness gradient rather than the absolute stiffness determines the direction and rate of axon growth (Koser et al., 2016). We hypothesize that when brain tissue is locally stiffened under pressure or pathological stimuli, a stiffness gradient is created with the surrounding tissue, which is sensed by Piezo1 in the neuronal axon, facilitating the extension of the axon in the direction of softer tissue.

### 3.3 Role of Piezo1 in microglia

#### 3.3.1 Piezo1 regulates microglial cell migration

Most macrophages, including microglia, migrate to the harder (denser) areas of the mechanical gradient, in a process called “durotaxis” (Blaschke et al., 2020). Recently, numerous studies have shown that Piezo1 channels are largely involved in mediating this durotaxis in microglia (Liu et al., 2021; Mchugh et al., 2010; Velasco‐Estevez et al., 2020). Hu et al. (2023) found that activation of Piezo1 channels in microglia mediated by stiff amyloid β (Aβ) fibrils promoted the migration and aggregation of microglia to Aβ plaques, whereas Piezo1 deficiency inhibited the migration and phagocytic activity of microglia. Jäntti et al. (2022) also showed that Yoda1‐mediated activation of Piezo1 promoted microglial migration to Aβ fibrils. However, a recent study showed that increased substrate stiffness significantly upregulated Piezo1 expression in microglia and inhibited cell migration, whereas knockdown of Piezo1 abolished this effect (Zhu et al., 2023). Liu et al. (2021) found that the use of GsMTx4 to inhibit the activation of Piezo1 channels at high glucose levels increased the migration rate of microglia. This is consistent with a study by Zhu et al. (2023), who found that activation of Piezo1 inhibited the migration of microglia to the chemoattractant, whereas inhibition of Piezo1 promoted cell migration. It would be interesting to further explore the dual roles of Piezo1 in regulating microglial migration.

Microglia are highly dynamic and once activated, they become adherent and migrate to sites of injury or inflammation (Mcwhorter et al., 2015). Piezo1 is involved in the regulation of cell migration through the regulation of cellular affinity for β1 integrins, Ca^2+^ influx, and actin dynamics (Mchugh et al., 2012; Mcwhorter et al., 2015; Zhu et al., 2023). Knockdown of Piezo1 decreases endogenous β1 integrin affinity at the plasma membrane, inhibits cell adhesion, and promotes cell migration in a non‐integrin‐dependent manner, which is responsible for increased tumor cell migration and metastasis in small‐cell lung carcinoma (Mchugh et al., 2012). However, cell lines stably overexpressing Piezo1 (hP1‐CL cell line) migrated approximately 10‐fold faster than HEK293T cells, whereas treatment with D‐GsMTx4 (5 μM) slowed cell migration more than 10‐fold (Maneshi et al., 2018). The finding of the latter study differs from integrin‐dependent cell migration and is associated with large Ca^2+^ influx mediated by Piezo1. Cytoskeletal integrity and plasticity determine the motility of cells under the influence of Piezo1 channels (Maneshi et al., 2018). Maneshi et al. (2018) showed that Piezo1‐mediated Ca^2+^ influx maintained cytoskeletal plasticity required for cell migration, whereas Piezo1 inhibitors GsMTx4 and Aβ_1–40_ peptide inhibited Ca^2+^ entry and increased cytoskeletal stress in hP1‐CL cells, thereby inhibiting cell migration. Piezo1 mediates Ca^2+^ influx, which leads to cytoskeletal changes that promote rapid and coordinated cell migration (Liu et al., 2021; Nourse & Pathak, 2017). Cell migration requires close control and coordination of actin dynamics, and activation of Piezo1 promotes actin polymerization, whereas knockdown significantly inhibits actin polymerization (Zhu et al., 2023). Thus, Piezo1 channels can regulate cell migration by regulating the affinity of integrins in cells, so that Piezo1 activation plays an inhibitory role in cell migration. In addition, Piezo1‐mediated massive Ca^2+^ influx is involved in regulating cell migration by modulating cytoskeletal plasticity, so that Piezo1 activation was often found to promote cell migration.

#### 3.3.2 Piezo1 regulates microglial phagocytosis

A characteristic property of microglia is their rapid activation in response to neuronal stress and their capacity for site‐targeted phagocytosis (Banati et al., 1993). Piezo1‐mediated mechanotransduction is essential for the phagocytosis of invading pathogens in disease‐associated microglia (DAM). Hu et al. (2023) showed that Piezo1 on the surface of microglia in a mouse model of AD senses Aβ fiber stiffness and promotes the phagocytic activity of microglia, thereby eliciting a protective response and limiting disease progression. Jäntti et al. (2022) injected Yoda1 intracranially into 5×FAD rats for 2 weeks and showed that Yoda1‐mediated Piezo1 activation promoted microglial phagocytosis and lysosomal activity to coordinate Aβ clearance. Recently, Schappe et al. (2018) showed that in TRPM7‐deficient mice and RAW 264.7 cells, lipopolysaccharide (LPS), or bacterial infection promotes reloading of the Piezo1‐toll‐like receptor 4 (TLR4) complex to remodel F‐actin and promote phagocytosis by macrophages, whereas knockdown of Piezo1 inhibits these responses. LPS stimulates TLR4 receptors to induce Piezo1‐mediated Ca^2+^ influx, thereby activating the CaMKII‐Mst1/2‐Rac axis to ingest and kill pathogens (Figure 2) (Geng et al., 2021).

A recent study showed that the phagocytic activity of microglia is regulated by omega‐3 fatty acids (*n* − 3 PUFAs) (Madore et al., 2020). In rodents, *n* − 3 PUFAs deficiency promotes microglia‐mediated phagocytosis of synaptic elements in the developing hippocampus (Madore et al., 2020). Previous studies have shown that PUFAs promote the inactivation of Piezo1 (Romero et al., 2019). Enhanced phagocytosis of microglia mediated by *n* − 3 PUFAs deficiency may be associated with the inhibition of Piezo1 inactivation. However, differentially expressed genes from RNA sequencing data suggested that Piezo1 in microglia may not be involved in the regulation of microglial cell function, because knockdown of Piezo1 in microglia did not change the regulation of *Trem2*, *Clec7a*, *Apoe*, or *Axl*, which are essential DAM marker genes that regulate the function of microglia (Hu et al., 2023).

#### 3.3.3 Piezo1 regulates the inflammatory phenotype of microglia

Mechanical signaling is inseparable from innate immunity (Solis et al., 2019). In addition to biological factors (e.g., IFN‐γ and LPS), physical factors (substrate stiffness and stretching) determine the innate function and phenotype of macrophages (Mcwhorter et al., 2015; Patel et al., 2012). As resident macrophages of the brain, microglia are usually divided into two mature phenotypes, respectively, termed classical activation (M1) and alternate activation (M2) (Mcwhorter et al., 2015). M1 is a pro‐inflammatory phenotype with high levels of tumor necrosis factor‐α (TNF‐α), interleukin (IL)‐12, and IL‐23 secretion. M2 is an anti‐inflammatory phenotype marked by the secretion of IL‐10, a known inhibitor of inflammation (Mcwhorter et al., 2015). According to the analysis of single‐cell RNA sequencing and single nuclei datasets, Piezo1 expression in microglia in AD is thought to be influenced by both cell clustering and racial differences (Grubman et al., 2019; Keren‐Shaul et al., 2017; Zhou et al., 2020). Piezo1 was downregulated in DAM in both 5×FAD mouse datasets, whereas being upregulated in the human AD‐specific subcluster M1 (Grubman et al., 2019; Keren‐Shaul et al., 2017; Zhou et al., 2020). Increased surface roughness and stiffness of the matrix tend to drive the activation of microglia toward the M1 phenotype and raise levels of the pro‐inflammatory substances IL‐1, IL‐6, and TNF‐α in the presence of LPS (Moshayedi et al., 2014; Refai et al., 2004; Zhu et al., 2023). It has been shown that the knockout of Piezo1 inhibits the release of pro‐inflammatory factors of microglia mediated by the stiff substrate in the presence of LPS (Zhu et al., 2023). However, further investigation is needed to determine whether Piezo1 is involved in regulating roughness‐mediated inflammatory cytokines in microglia.

The role of Piezo1 in the regulation of the inflammatory response of immune cells is cell type‐specific. In the brain, in the presence of inflammation‐stimulating factors (Aβ or LPS), activation of Piezo1 tends to suppress the pro‐inflammatory phenotype of glial cells. Malko et al. (2023) showed that 0.3–3 μM Yoda1 inhibited LPS‐induced microglial activation and production of the pro‐inflammatory factors TNF‐α and IL‐6, which could be abolished by GsMTx4 or si*Piezo1*RNA. Activation of Piezo1 exerts an anti‐inflammatory effect through inhibition of the NF‐κB pathway, but not the extracellular signal‐regulated kinase (ERK) and p38 pathways (Malko et al., 2023). Consistent with this, Liu et al. (2021) showed that inhibition of Piezo1 promotes LPS‐mediated secretion of pro‐inflammatory cytokines from microglia. In addition, Yoda1‐mediated activation of Piezo1 in astrocytes has been shown to inhibit the LPS‐induced release of pro‐inflammatory factors (TNF‐α and IL‐6, IL‐1β) (Velasco‐Estevez et al., 2020). However, a very recent study by Zhu et al. (2023) showed that the addition of Yoda1 (10 μM) increased the mRNA expression levels of IL‐1β, IL‐6, and TNF‐α in BV2 cells stimulated with LPS. This is in stark contrast to the study by Malko et al., and we speculate that the difference in Yoda1 concentration may be responsible for the difference in experimental conclusions, as the other experimental conditions were almost identical. However, activation of the immune cell Piezo1 often mediates pro‐inflammatory effects outside the CNS (Liu et al., 2022; Solis et al., 2019). Bone marrow‐derived macrophages (BMDMs) show high expression of Piezo1, and knockdown of Piezo1 completely eliminates cyclic hydrostatic pressure‐mediated upregulation of pro‐inflammatory genes (*Il1b*, *Cxcl10*, and *Ptgs2*) in BMDMs (Solis et al., 2019).

Recent studies on the role of Piezo1 in regulating the inflammatory phenotype of microglia were nearly conducted in vitro (Liu et al., 2021; Malko et al., 2023). Thus, further research is required to determine how Piezo1 activation affects the inflammatory phenotype of microglia in vivo. Furthermore, it would be of interest to further explore the role of Yoda1 concentrations on the regulation of microglial inflammatory phenotypes. These findings may open new possibilities for controlling microglia in neurodegenerative diseases and developing new therapeutic strategies.

### 3.4 Role of Piezo1 in astrocytes

Due to their complex and delicate morphology, astrocytes are highly mechanosensitive and sense local environmental cues through mechanoreceptors (Procès et al., 2022). Hippocampal astrocytes actively regulate adult neurogenesis both by promoting proliferation of adult NSCs and by controlling neuronal fate (Song et al., 2002). A recent groundbreaking study showed that Piezo1 on hippocampal astrocytes responds to mechanical stimuli and plays an irreplaceable role in neurogenesis, NSC proliferation, and cognitive function (Chi et al., 2022). The research shows that Piezo1 activation induces significant cationic currents and transient Ca^2+^ influx in astrocytes, which promotes the release of adenosine triphosphat (ATP) to promote adult neurogenesis in the dentate gyrus (DG) (Figure 2) (Chen & Qiu, 2022). The authors found that deletion of the Piezo1 gene in astrocytes leads to reduced hippocampal DG volume, brain weight, and NSC proliferation and impairs long‐term potentiation in mice, which could be rescued by ATP (Chi et al., 2022). In addition, Piezo1 overexpression in astrocytes enhanced learning and memory (Chi et al., 2022). This study highlights the important role of Piezo1‐mediated astrocyte mechanotransduction in regulating neurodevelopment. However, the specific mechanical stimuli that activate the Piezo1 channel during brain development remain unclear.

Under normal physiological conditions, astrocytes support and promote neuronal survival (Sofroniew & Vinters, 2010), whereas CNS infection, injury, or disease induces activation of “resting astrocytes” to “reactive astrocytes,” characterized by increased expression of intermediate filaments, such as glial fibrillary acidic protein (Liddelow et al., 2017). Resting astrocytes rarely express Piezo1, whereas Piezo1 expression was significantly upregulated in reactive astrocytes (Chi et al., 2022; Velasco‐Estevez et al., 2020, 2018). There are two subpopulations of reactive astrocytes: A1 and A2. A1 astrocytes are a neurotoxic subtype of reactive astrocytes that drive neuroinflammation, whereas A2 mediates neuroprotective effects (Liddelow et al., 2017). However, it is unclear whether the upregulation of Piezo1 differs between A1 and A2 reactive astrocyte subpopulations. Mechanical stimulus‐mediated Ca^2+^ currents are known to be involved in the regulation of astrocyte responsiveness (Scemes & Giaume, 2006). However, whether Piezo1 channel–mediated Ca2+ currents are involved in the regulation of astrocyte activation needs to be further explored.rAlhough Liu et al. (2021) found that the Yoda1‐mediated activation of Piezo1 on optic nerve head (ONH) astrocytes induce a reactive phenotype. It is important to investigate Whether inhibition or deletion of Piezo1 affects the activation of astrocytes in order to obtain a deeper understanding of the functionality of Piezo1 channels in astrocyte activation .

In addition, Piezo1 on astrocytes is also involved in the regulation of the inflammatory response. Specific stimuli (including Aβ_1–42_ and LPS) but not all cellular stressors upregulate astrocytic Piezo1 expression (Velasco‐Estevez et al., 2018, 2020). Yoda1‐mediated Piezo1 activation increases intracellular Ca^2+^ oscillations and inhibits the production and release of pro‐inflammatory cytokines (e.g., IL‐1β and TNFα) and C‐X3‐C motif chemokine 1 from LPS‐activated astrocytes (Velasco‐Estevez et al., 2020). Activation of astrocytic Piezo1 may inhibit neuroinflammation, consistent with the findings in microglia (Liu et al., 2021). Interestingly, GsMTx4 had no or little effect on the release of pro‐inflammatory factors from reactive astrocytes, and the anti‐inflammatory effect of Yoda1 in reactive astrocytes was not entirely dependent on the activation of Piezo1 (Velasco‐Estevez et al., 2020). Piezo1 is known to regulate the expression of the mechanosensitive transcriptional cofactor YAP (Pathak et al., 2014; Wan et al., 2023), which is highly expressed in astrocytes and inhibits astrocyte reactivity by downregulating the pro‐inflammatory JAK‐signal transducers and activators of transcription (STAT) pathway (Huang et al., 2016). Thus, the anti‐inflammatory effect of Yoda1 may depend on YAP‐mediated downregulation of the JAK‐STAT pathway. It is worth mentioning that Piezo1 on ONH astrocytes is involved in the regulation of cell proliferation by regulating the nuclear localization of YAP and cell cycle‐related factors, including cyclin D1 and c‐Myc (Wan et al., 2023).

Astrocytes are the most abundant cells in the brain, and they have distinct regional properties. The mechanoreceptors on astrocytes in different regions are perhaps responsible for different functions (Song et al., 2002). For example, Piezo1 on astrocytes in the cortical and hippocampal regions is involved in the regulation of neurogenesis (Chi et al., 2022), whereas the same channels located on ONH astrocytes are involved in regulating astrocyte reactivity and proliferation (Liu et al., 2021). Notably, Piezo1 expression in brain astrocytes is higher than that of other mechanoreceptors (e.g., transient receptor potential vanilloid 4 [TRPV4] and transient receptor potential ankyrin 1) (Chi et al., 2022), suggesting an important role for Piezo1 in astrocytic mechanotransduction. However, there are limited studies on the function of Piezo1 in astrocytes. The effects of Piezo1 are dependent on Ca^2+^ signaling and downstream effectors such as ATP (Chi et al., 2022) and YAP (Pathak et al., 2014; Wan et al., 2023). However, other effectors downstream of Piezo1‐mediated Ca^2+^ signaling in astrocytes need to be further explored. In addition, it is necessary to clarify the relationship between Piezo1‐mediated mechanotransduction on astrocytes and disease, as well as the mechanistic link between microglia and astrocytes.

### 3.5 Roles of Piezo1 in oligodendrocytes and Schwann cells

Similar to astrocytes, oligodendrocytes provide nutritional and metabolic support for neurons (Nave, 2010) and also protect neurons from damage by forming myelin sheaths that encase neuronal axons in the external mechanical environment (Marton et al., 2019). Oligodendrocytes and oligodendrocyte progenitor cells (OPCs) express the mechanoreceptor Piezo1 and are highly sensitive to mechanical stimuli (e.g., topological variations) (Segel et al., 2019; Velasco‐Estevez et al., 2022; Webb et al., 1995). Previous studies have shown that impaired OPC recruitment and subsequent differentiation are directly related to the age‐related reduction in the efficiency of myelin regeneration (Sim et al., 2002). However, Segel et al. (2019) showed that the decreased ability of OPCs to proliferate and differentiate in aging brains is attributed to “niche” stiffness. The stiff niche is sensed by the mechanoreceptor Piezo1, thereby inhibiting the ability of OPCs to proliferate and differentiate (Figure 2) (Segel et al., 2019). Piezo1 knockdown eliminates OPC perception of the external environment and rescues stiff substrate‐induced inhibition of OPC viability (Segel et al., 2019). A recent study showed that in vitro activation of Piezo1 inhibits oligodendrocyte proliferation and migration, and as oligodendrocytes mature, Piezo1 expression decreases (Velasco‐Estevez et al., 2022). However, in vivo, data from Segel et al. (2019) showed that Piezo1 expression was significantly higher in the OPCs of aged rats than those of neonatal rats. Thus, age‐related changes in oligodendrocytic Piezo1 expression may not be linear.

Schwann cells are the peripheral counterparts of oligodendrocytes and are involved in the growth, regeneration, and nutrition of peripheral nerves. A recent innovative study has shown that Piezo1 and Piezo2 are among the most widespread mechanosensitive ion channels expressed by Schwann cells, and both of them are synergistically involved in the regulation of myelination (Acheta et al., 2022). In Piezo1 knockout sciatic nerves, disruption of Piezo‐mediated transient Ca^2+^ currents promoted the activation of TAZ, phosphoinositide 3‐kinase (PI3K)/AKT, and mitogen‐activated protein kinase (MAPK)/ERK, as well as the phosphorylation and inactivation of YAP, thereby promoting myelin formation (Acheta et al., 2022). Furthermore, it has been proposed that Piezo2 is required for proper myelin thickening during early development (Acheta et al., 2022). Oligodendrocytes and Schwann cells are involved in the formation of myelin sheaths in axons and are closely associated with demyelinating diseases. The roles of Piezo1 in oligodendrocytes and Schwann cells are described in detail in Section 4.2.

In general, all brain cells including glial cells and neurons are mechanosensitive and express the Piezo1 mechanoreceptor. During brain development or disease, mechanistic signals will be translated into intracellular signals via Piezo1 to control key neurobiological processes (Table 1).

## 4 PIEZO1‐MEDIATED MECHANOTRANSDUCTION IN CNS DISEASE

A key hallmark of CNS disease is the change of tissue stiffness due to inflammation and scarring (Ong et al., 2020). Altered mechanical properties of the brain are an essential manifestation and aggravating factor of many neurodegenerative diseases (Tyler, 2012). A large number of studies have demonstrated the involvement of Piezo1‐mediated mechanotransduction in the pathophysiology of aging, brain injury, and neurodegenerative diseases (Chen et al., 2018; Jäntti et al., 2022; Qu et al., 2023; Segel et al., 2019; Wang et al., 2019). Below we summarize the roles of Piezo1 in mediating brain disease processes.

### 4.1 Role of Piezo1 in aging

As they age, all multicellular organisms experience a reduction in tissue and organ function, which is thought to be primarily caused by the loss of stem cell (SC) viability and differentiation capacity over time (Wang et al., 2021). There is an emerging view that age‐related stiffening of the SC niche (i.e., the microenvironment in which SCs survive and self‐renew) is a major contributor to impaired SC viability (Gopinath & Rando, 2008; Koester et al., 2021; Ryu et al., 2021; Segel et al., 2019). SCs of different origins appear to exhibit similar niche stiffness and functional impairment with age. For example, Koester et al. (2021) showed that aging‐related niche stiffness suppressed the transcriptional activity of genes associated with self‐renewal and differentiation in mouse hair follicle SCs, thereby promoting cellular senescence. The aforementioned niche stiffness inhibited the proliferation, migration, and differentiation abilities of brain OPCs (Segel et al., 2019). Increasing cortical and hippocampal tissue stiffness in rats from infancy to maturity is correlated with a specific pattern of hippocampal NSC‐mediated neurogenesis (Ryu et al., 2021). Muscle‐specific SC niche aging inhibits SC activity and proliferation and promotes muscle senescence (Gopinath & Rando, 2008). Overall, microenvironmental niche mechanisms, rather than intracellular factors, may be the main determinant of aging in adult SC systems. Numerous atomic force microscopy (AFM) results also confirmed that brain aging is accompanied by changes in the mechanical properties of brain tissue, as evidenced by an increase in stiffness with age (Elkin et al., 2010; Javier‐Torrent et al., 2021; Ryu et al., 2021; Segel et al., 2019).

Restoration of the senescent niche of aging SCs or inhibition of their perception of the sclerotic matrix can restore SC function (Koester et al., 2021; Segel et al., 2019). Recent reliable evidence suggests the mechano‐gated Piezo1 channel is required for cellular perception of senescence‐mediated sclerotic substrates (Segel et al., 2019). Piezo1 mechanoreceptors have previously been shown to be involved in the perception of substrate stiffness by NSCs and the modulation of their spectral selection (Pathak et al., 2014). Moreover, Piezo1 was found to be highly expressed in the rat cerebellar ventricular axis, hippocampal fibers, axonemal tracts, and medulla of the brainstem, and its expression increased with age in the hippocampus and cortex (Velasco‐Estevez et al., 2018). There is direct evidence that Piezo1 channels are specialized mechanosensor that conducts niche mechanical cues in brain OPCs, and Piezo1 knockdown rescues the decrease of OPC viability induced by the aging microenvironment (Segel et al., 2019). Investigating the mechanism of Piezo1‐mediated tissue mechanotransduction could be a key factor in the success of cell‐based therapies for age‐related diseases. Given that aging‐mediated Piezo1 upregulation is primarily seen in the rat prefrontal cortex, hippocampal CA1, and DG, all of which are important for information processing and memory (Velasco‐Estevez et al., 2018), further research is needed to determine whether Piezo1 channels are involved in aging‐related cognitive dysfunction.

Several studies have provided partial explanations for mechanically mediated Piezo1 activation to promote aging outside the CNS. During aging, the nucleus pulposus (NP) underwent a transition from “fluid” to “solid,” and the stiffness of the ECM also increased (Iatridis et al., 1997; Wang et al., 2021). Piezo1 knockdown protected human NP cells from oxidative stress‐induced senescence and apoptosis on a sclerotic ECM (Wang et al., 2021). Intracellular Ca^2+^ accumulation is a common phenomenon in aging cells, causing mitochondrial dysfunction and reactive oxygen species (ROS) production, thereby promoting the aging process (Wiel et al., 2014). Activation of Piezo1 in chondrocytes by Yoda1 inhibited proliferation and promoted aging‐related secretory phenotypes, while also increasing cellular Ca^2+^ and ROS concentrations (Ren et al., 2022). Furthermore, Piezo1 activation is involved in the ferroptosis pathway, which promotes cellular senescence (Chen et al., 2021). Inhibition of Piezo1 channel activity increased glutathione peroxidase 4 expression and attenuated the ferroptosis phenotype in a mouse model of osteoarthritis (Chen et al., 2021). Mechanical stress accelerates the aging of NP cells by activating Piezo1 and triggering positive feedback from NF‐κB and periostin (Wu et al., 2022). Therefore, therapies targeting Piezo1 may be a viable strategy to slow down aging and ameliorate the adverse changes of the body during aging in the future.

### 4.2 Role of Piezo1 in demyelinating diseases of the CNS

Demyelination diseases are characterized by a loss of the myelin sheath while the axon is relatively intact, and demyelination of nerve fibers is the main pathological feature (Franklin & Ffrench‐Constant, 2008). The increased overall stiffness of brain tissue is the main mechanical feature of the chronic progression of demyelination (Pyka‐Fościak et al., 2020; Urbanski et al., 2019). Oligodendrocytes and Schwann cells are responsible for the formation of myelin sheaths in the central axis of the CNS and peripheral neurites, respectively. Several investigations have indicated that Schwann cells and oligodendrocytes are sensitive to mechanical cues such as matrix stiffness and roughness (Rosso et al., 2019; Segel et al., 2019; Unal et al., 2020; Webb et al., 1995). Accordingly, changes of substrate stiffness affect the differentiation and proliferation of oligodendrocytes and Schwann cells (Rosso et al., 2019). However, Schwann cells with dense vimentin filaments have greater resistance to deformation, suggesting that the mechanical resistance of adult Schwann cells protects the neurons they encase (Rosso et al., 2019).

**FIGURE 3:**
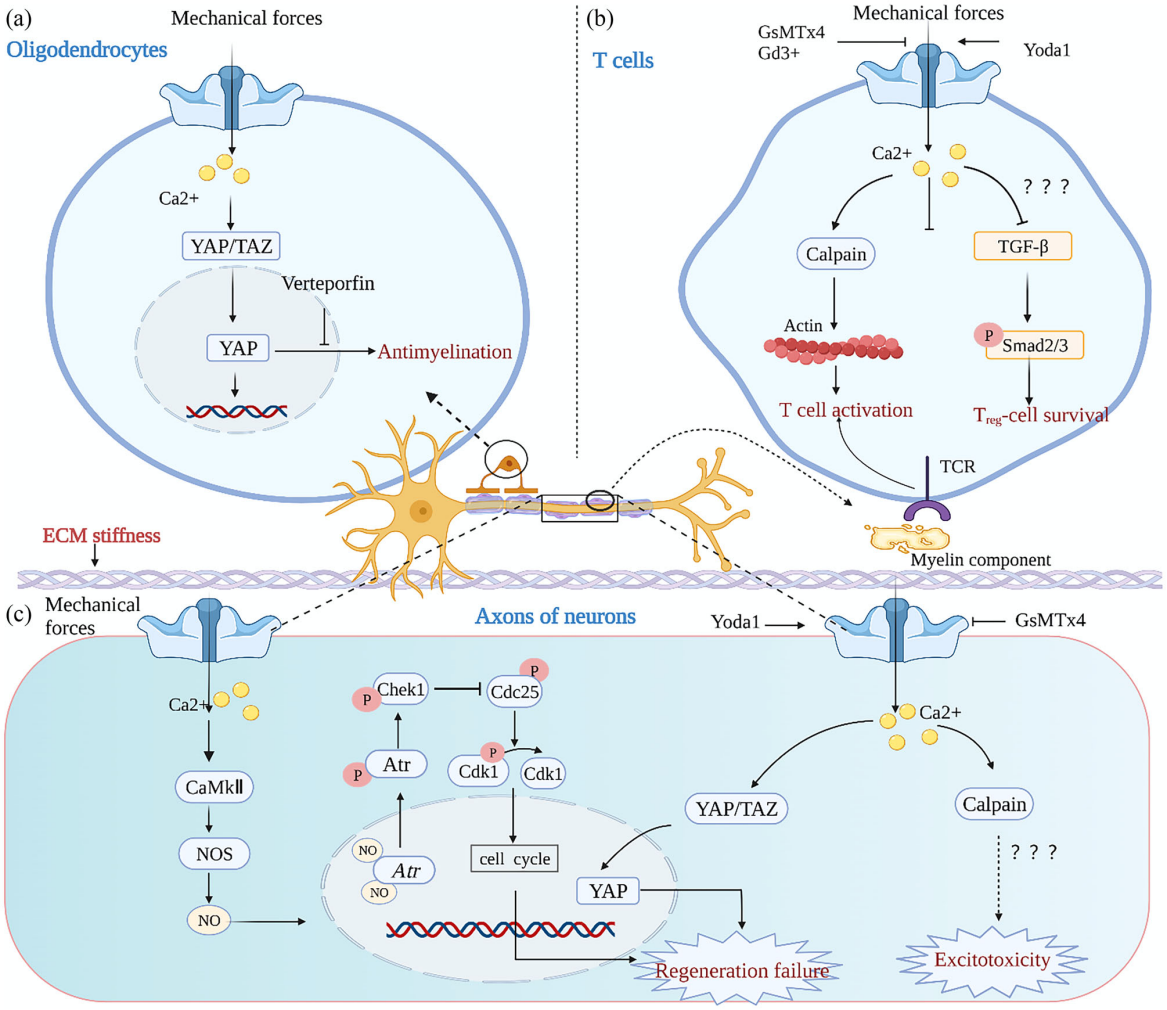
**Schematic diagram of the role of Piezo1 in demyelinating diseases**. (a) The Piezo1/yes‐associated protein (YAP)/binding motif (TAZ) pathway regulates oligodendrocyte myelination. Mechanical forces activate Piezo1 on oligodendrocytes, causing transient Ca^2+^ currents, activating the YAP/TAZ pathway, and encouraging the nuclear localization of YAP, which is activated and limits myelin regeneration. This effect can be inhibited by the YAP inhibitor verteporfin. (b) Piezo1‐mediated mechanotransduction regulates T/regulatory T cell (Treg) cell activity. In autoreactive effector T cells, the mechanical force causes Piezo1 to open and allow Ca^2+^ influx, which activates calpain. Activated calpain reorganizes the actin structure, thus facilitating the optimal activation of T cells. In Tregs cells, mechanical activation of Piezo1 inhibits the TGF‐β/Smad2/3 pathway, resulting in reduced viability. Suppression of Treg cell proliferation and activity exacerbates the autoimmune response in demyelinating diseases. (c) The role of Piezo1 in axonal regeneration. Upon mechanical stimulation, Piezo1 channels open to promote Ca^2+^ influx, which then leads to the generation of NO as a second messenger. Then, NO diffuses to the nucleus to activate Atr and its associated complexes, initiating the activation of the Atr/Chek1 axis. Chek1 negatively regulates Cdc25 to dephosphorylate and activate Cdk1. Finally, phosphorylated and inactive Cdk1 leads to regeneration failure. Mechanical stress or Yoda1‐mediated activation of Piezo1 promotes Ca^2+^ inward flow and activates calpain. Activation of calpain signaling is known to lead to neurodegeneration (Wang et al., 2019). Thus, Piezo1 activation may promote axon demyelination through excitotoxicity mediated by calpain activation.

Piezo1 plays an essential role in oligodendrocyte‐mediated myelin formation and regeneration. At the beginning of myelin formation, when oligodendrocytes begin to surround axons, Piezo1 on the axonal surface of neurons senses the mechanical forces generated by myelinated oligodendrocytes wrapped around the axonal surface and participates in regulating myelin formation (Velasco‐Estevez et al., 2020). Numerous studies have shown that Piezo1 is a negative regulator of myelin formation in oligodendrocytes and Schwann cells (Acheta et al., 2022; Li et al., 2021; Velasco‐Estevez et al., 2020). Piezo1 is highly active after axonal injury and during axonal regeneration (Segel et al., 2019; Velasco‐Estevez et al., 2020), which may exacerbate nerve damage and demyelination. In brain tissue sections and cellular models of demyelination, the Piezo1 inhibitor GsMTx4 has been shown to inhibit chemically induced neuronal demyelination and neuronal damage, whereas Yoda1 directly induced axonal damage and demyelination in Purkinje neurons (Velasco‐Estevez et al., 2020). GsMTx4 promotes myelin formation by increasing the level of phosphorylated neurofilament heavy chain (Velasco‐Estevez et al., 2020). Notably, GsMTx4 not only blocks Piezo1 but also some voltage‐gated sodium and potassium channels such as transient receptor potential channel 1 and TRPC6 (Gottlieb et al., 2007). Currently, there is no direct evidence for the role of Piezo1 channel activation in regulating axonal damage and demyelination. A recent study found that activation of YAP contributed to the failure of myelin regeneration induced by a stiff mechanical microenvironment and inhibited the myelination of oligodendrocytes (Ong et al., 2020). Previously, Pathak et al. (2014) showed that activation of Piezo1 channels induces transient Ca^2+^ currents and promotes the nuclear localization of YAP in hNSPCs. Furthermore, knockdown of Piezo1 promotes YAP inactivation and facilitates myelin regeneration in peripheral Schwann cell‐mediated myelin regeneration (Acheta et al., 2022). Therefore, we hypothesize that in demyelinating diseases, activation of Piezo1 due to a rigid matrix activates YAP and promotes its nuclear translocation, thereby inhibiting oligodendrocyte‐mediated myelin regeneration (Figure 3a).

Multiple sclerosis (MS) is the most common demyelinating disease, manifesting as demyelinating lesion in the white matter of the CNS (Reich et al., 2018). In addition to the direct role of Piezo1 in myelin regeneration mentioned above, Piezo1 is also involved in the regulation of MS‐related immune responses. In experimental allergic encephalitis (EAE; an in vivo murine model of MS), knockdown of Piezo1 in T cells ameliorated disease severity (Jairaman et al., 2021). This result was partly attributed to the fact that the deletion of Piezo1 in T cells activates the TGF‐β signaling pathway and promotes Smad2/3 phosphorylation, thereby promoting regulatory T cell (Treg) activity (Figure 3b) (Jairaman et al., 2021). Activated Tregs suppress autoimmune responses in MS/EAE and limit inflammation in EAE mice (Figure 3b) (Jairaman et al., 2021). It has been proposed that Piezo1‐mediated mechanotransduction contributes to T‐cell activation. Mechanical stretching during immunological synapse formation triggers Piezo1 activation and Ca^2+^ influx, which activates calpain to promote TCR signaling and T‐cell activation (Figure 3b) (Liu et al., 2018). Thus, activation of Piezo1 during demyelination may exacerbate the progression of MS by promoting the activation of autoimmune T cells (Figure 3b). Notably, Piezo1 mRNA was downregulated in the white matter of MS brains compared to healthy controls (Velasco‐Estevez et al., 2022). This downregulation of Piezo1 in the MS brain is not specific to demyelinated or degenerated brain regions but rather to the whole brain (Velasco‐Estevez et al., 2022). However, there are no reliable studies to explain this downregulation of Piezo1 in MS brains, and given the inhibitory effect of Piezo1 on axon formation, we hypothesize that this may be attributed to feedback regulation of blocked axon and myelin regeneration.

Inhibition of myelin regeneration has profound effects on axonal health, whereas irreversible loss of axons promotes the progression of demyelinating diseases (Franklin & Ffrench‐Constant, 2008). Recently, Li et al. proposed that Piezo1 can inhibit axon regeneration through the NOS‐Atr‐Chek1‐Cdc25 axis (Li et al., 2021; Song et al., 2019). In detail, activation of the Atr‐Chek1 pathway was found to be involved in inhibiting axon regeneration of sensory neurons in a *Drosophila* model of sensory neuron injury and a mouse model of sciatic neuropathy, whereas osmotic stress‐mediated activation of the Piezo1 pathway and its downstream NO signaling are key factors contributing to the activation of the Atr‐Chek1 pathway (Li et al., 2021). In addition, the chemical agonist of Piezo1, Yoda1, has a weak inhibitory effect on axonal regeneration after injury via the CamKII‐NOS‐PKG pathway (Song et al., 2019). In NSCs, mechanical activation of Piezo1 channels leads to Ca^2+^ influx and nuclear localization of the YAP/TAZ transcriptional coactivators (Pathak et al., 2014), which are also involved in regulating myelin formation in oligodendrocytes (Shimizu et al., 2017) and Schwann cells (Grove et al., 2017). In addition, the YAP/TAZ axis integrates intracellular biochemical and mechanical signals, inhibits axonal regeneration by driving the Hippo signaling pathway, and controls the expression of the α6 integrin subunit (Figure 3c). Piezo1 localized on the ER recruits the small GTPase R‐Ras to relocate to the ER, thereby maintaining integrin activation and subsequent calpain activation (Mchugh et al., 2010). Excessive activation of Piezo1 may lead to excitotoxicity and CNS axon demyelination via calpain‐mediated destabilization of integrin signaling (Figure 3c) (Velasco‐Estevez et al., 2020; Wang et al., 2019).

### 4.3 Role of Piezo1 and Aβ‐mediated mechanical signaling in AD

AD is the most common progressive neurodegenerative disease. Continuous changes in the mechanical environment of the brain are present throughout the disease process (Murphy et al., 2016). Extracellular Aβ plaque deposition and intracellular neurofibrillary tangles are the main pathological features of AD (Mckhann et al., 1984). Aβ is a rigid extracellular protein aggregate that is more than 10^6^ times stiffer than normal brain tissue (Aguzzi & O'connor, 2010), thus directly affecting brain tissue stiffness and roughness (Blumenthal et al., 2014). A clinical study showed that in early AD, brain stiffness increased with Aβ deposition when only Aβ positivity was found without cognitive impairment, whereas brain imaging in patients with advanced AD showed a decrease in brain stiffness with Aβ deposition (Murphy et al., 2011, 2016). It is hypothesized that the deposition of Aβ is the main cause of increased brain stiffness in early AD patients, whereas in the late stage of AD, Aβ oligomerizes to form toxic fibrils and amyloid plaques, which induce downstream pathological events including neurofibrillary tangles caused by tau kinase dysregulation and oxidative stress, which ultimately lead to massive synapse loss and neuronal cell death (Hardy & Selkoe, 2002). This in turn explains why the brain becomes soft in the late stages.

Piezo1, a channel protein that senses changes in the mechanical environment of the brains of AD patients, was originally found to be upregulated in reactive astrocytes surrounding amyloid plaques in AD patients (Satoh et al., 2006). Similarly, in the 18‐month‐old TgF344‐AD rat, Piezo1 was upregulated in reactive astrocytes surrounding Aβ plaques compared to astrocytes outside the plaques (Velasco‐Estevez et al., 2018). Notably, Piezo1 on astrocytes appears to respond specifically to Aβ, whereas neuroinflammatory cellular stressors (such as IL‐17A, hydrogen peroxide (H_2_O_2_), or TNF‐α) have no effect on the expression of Piezo1 on astrocytes (Velasco‐Estevez et al., 2018). However, Maneshi et al. (2018) found that Aβ monomer inhibited Piezo1 at femtomolar to picomolar concentrations under mechanical stimulation by fluid shear stress, whereas the peptide form of Aβ aggregates was much less potent against Piezo1. In AD brains, neurons perceive the higher roughness of amyloid plaque aggregates and mediate the harmful effects of the rough environment through Piezo1 (Blumenthal et al., 2014). Conversely, the Piezo1 cation channel inhibitor GsMTx4 eliminates the ability of neurons to perceive the environment and promotes the beneficial effects of uncoupling neurons from astrocytes (Blumenthal et al., 2014). Taken together, Piezo1 may be a key mediator of the response to Aβ in AD brains, in both glial cells and neurons.

**FIGURE 4:**
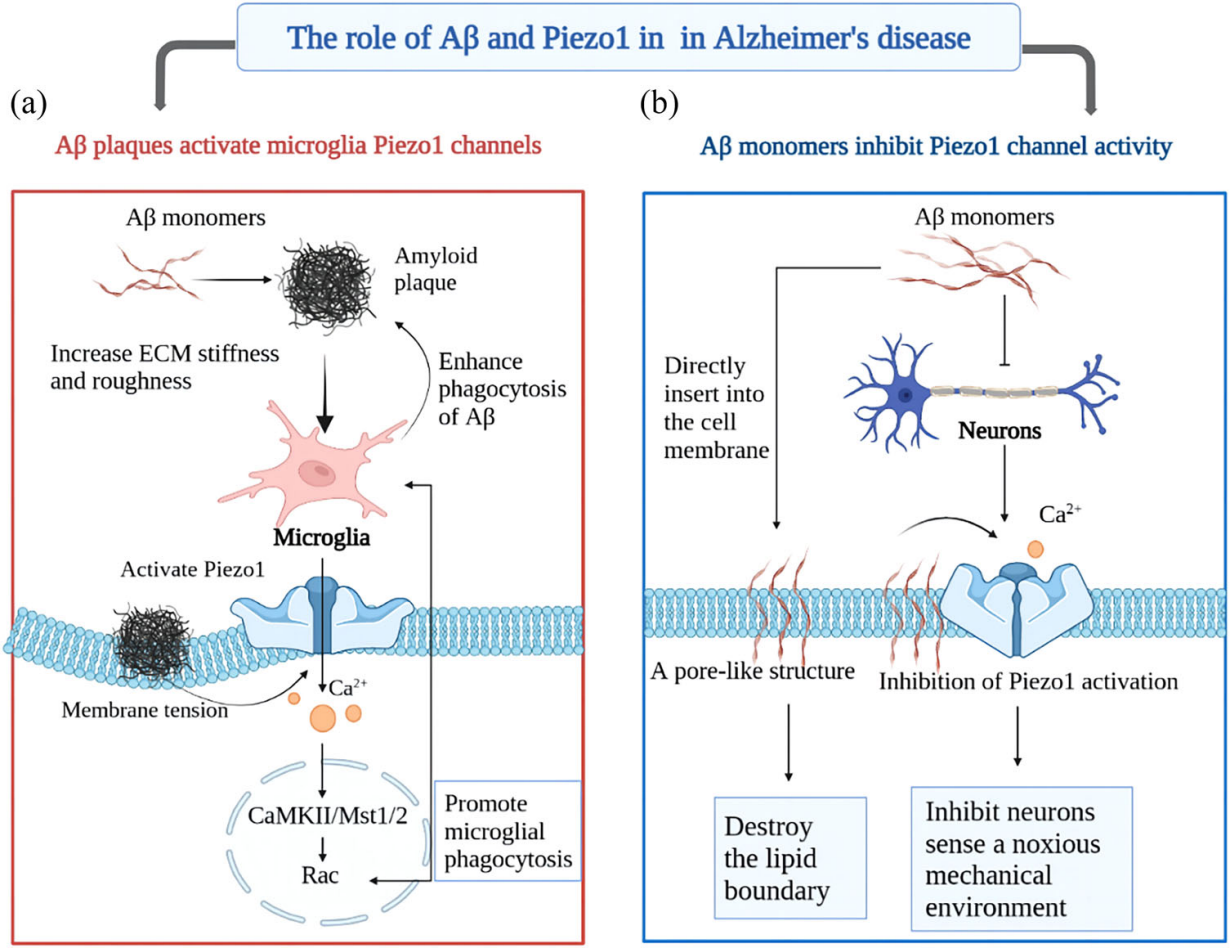
**Different roles of Piezo1 in mediating the interaction of amyloid β (Aβ) with the cell membrane in two types of cells**. (a) Monomeric Aβ precipitates and aggregates to form stiff and rough amyloid plaques. Microglia sense the rough environment of amyloid plaques via mechanosensitive Piezo1. Amyloid plaques activate Piezo1 channels and promote Ca^2+^ influx by altering membrane tension, which in turn promotes microglial phagocytosis of amyloid plaques by activating the CaMKII‐Mst1/2‐Rac axis. (b) The Aβ monomers directly insert into the cell membrane and destroy the lipid boundary of Piezo1, thus inhibiting the ability of Piezo1 to respond to mechanical forces. This process may block the deleterious effects of the stiff and rough mechanics of the AD brain on neurons and exert neuroprotective effects. ECM, extracellular matrix.

This raises the question of how Aβ specifically acts on Piezo1. First of all, Aβ is known to be a polypeptide with amphipathic properties that both interact with membrane proteins and alters the inherent mechanical properties of the lipid bilayer of the cell membrane (e.g., membrane curvature, membrane tension, and elastic modulus) (Lundbæk et al., 2010; Soreghan et al., 1994). The mechanosensitive cation channel Piezo1 is gated by sensing membrane tension and curvature. Exogenously added Aβ monomers were found to co‐localize with Piezo1 in hP1‐CL (Maneshi et al., 2018). Both Aβ and Piezo1 are concentrated in the structural domains of cholesterol‐rich lipid rafts (Kawarabayashi et al., 2004; Poole et al., 2014). Therefore, there are three possible mechanisms of Aβ action on Piezo1. First, the stiff Aβ peptide may bind to the cell membrane surface, compresses the cell membrane (the membrane collapse pressure of pure Aβ_1–42_ peptide is ∼33 mN/m), and alters the membrane mechanics (Figure 4) (Ambroggio et al., 2005; Markin & Sachs, 2015; Niu et al., 2018). This in turn alters the state of Piezo1, which often results in activation. Second, the Aβ monomers may directly insert into the cell membrane and destroy the lipid boundary of Piezo1 (Lundbæk et al., 2010), thus inhibiting the ability of Piezo1 to respond to mechanical forces (Figure 4). Third, Aβ may inhibit the activation of Piezo1 channels by perturbing the lipid structure. Aβ peptide is known to be concentrated in lipid rafts (Kawarabayashi et al., 2004), and the very low concentrations of monomeric Aβ required to inhibit channel function may be concentrated in the bound structural domain containing Piezo1 (Maneshi et al., 2018). Irreversible membrane disruption and Ca^2+^ leakage induced by soluble Aβ oligomers are the prevalent mechanisms of its neurotoxic effects (Ambroggio et al., 2005; Demuro et al., 2005). Aβ oligomers have neurotoxic effects, whereas Aβ monomers at physiological concentrations are neuroprotective, improve synaptic plasticity, and promote neuronal survival (Giuffrida et al., 2009; Parihar & Brewer, 2010). The different effects of Aβ monomers and Aβ oligomers on Piezo1 channels described above may be attributed to different Aβ forms interacting with Piezo1 through different mechanisms. Whether Aβ monomers or oligomers activate Piezo1 by altering membrane mechanics or directly inhibit Piezo1 through peptide–protein interactions deserves further in‐depth exploration.

**FIGURE 5:**
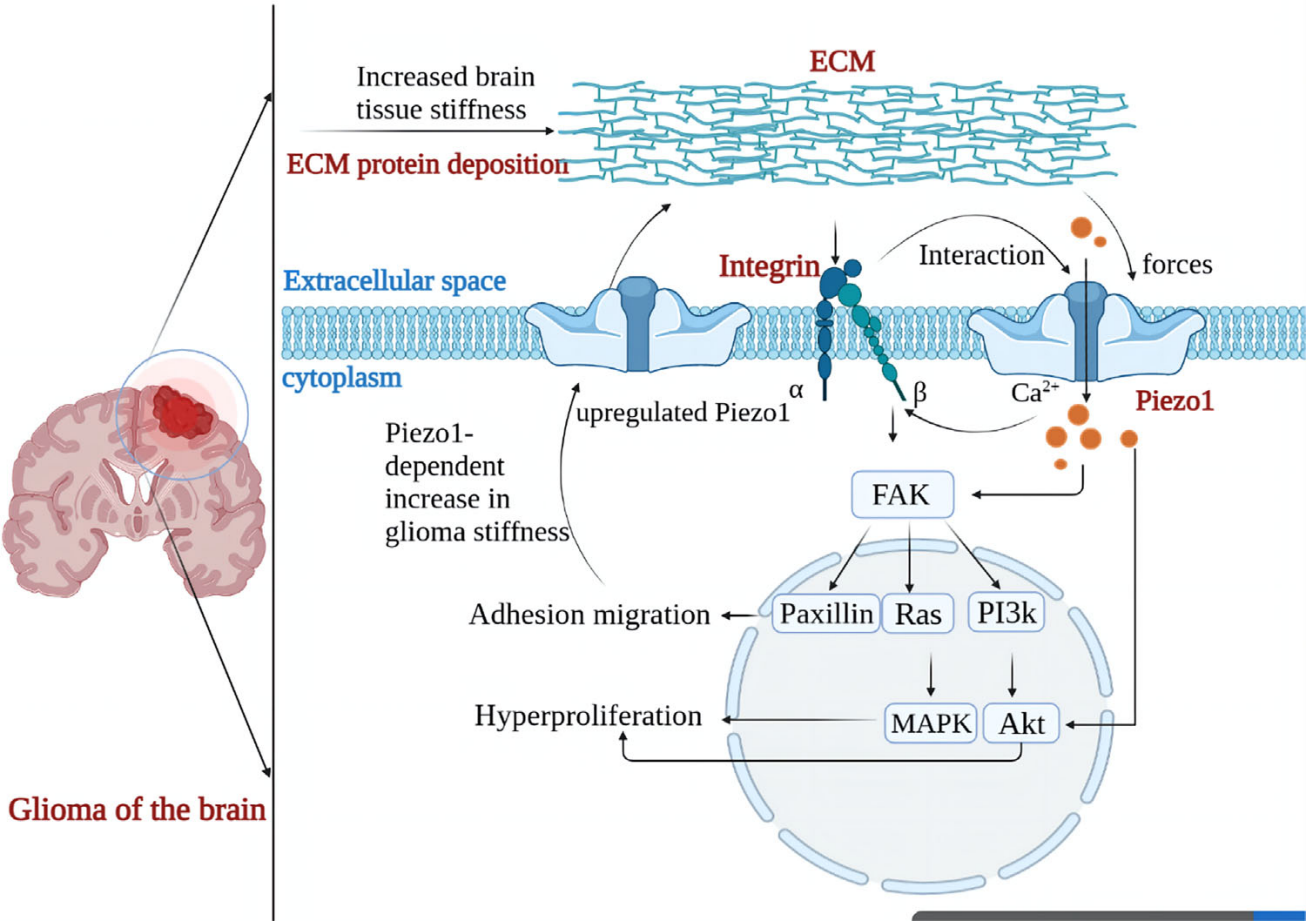
P**athological processes mediated by the mechanosensitive channel protein Piezo1 in gliomas**. First, the deposition of large amounts of extracellular matrix (ECM) proteins increases tissue stiffness near the glioma lesion. Consequently, the mechanical environment mediated by ECM protein deposition activates the Piezo1 channel to promote Ca^2+^ influx, which in turn activates the integrin‐dependent kinase (FAK) signaling pathway or directly activates the Akt pathway to promote tumor cell migration and proliferation. In addition, Piezo1 interacts with integrin‐FAK signaling and participates in the regulation of ECM remodeling, promoting the expression of Piezo1 channel proteins, which in turn further increases tissue stiffness and tumor cell proliferation. In this way, a positive feedback loop is formed to promote the pathological progression of glioma.

In addition to its role in astrocytes, Geng et al. (2021) showed that Piezo1 is also involved in the Aβ‐ and LPS‐mediated inflammatory activation of macrophages (Figure 5). As described above, TLR4, the receptor for Aβ and LPS, co‐localizes with Piezo1, and Piezo1 regulates macrophage phagocytosis by coordinating TLR4 signaling and inducing Ca^2+^ influx to activate the CaMKII‐Mst1/2‐Rac axis (Geng et al., 2021). In agreement with Geng et al., Jäntti et al. recently found that Yoda1‐mediated activation of Piezo1 promotes the survival, phagocytosis, and lysosomal activity of human‐induced pluripotent SC‐derived microglia‐like cells, which in turn increases the clearance of Aβ (Jäntti et al., 2022). In addition, the rough and stiff environment provided by amyloid plaques may upregulate Piezo1 in microglia, thereby promoting their phagocytic activity (Figure 4) (Hu et al., 2023). Overall, Piezo1 in microglia perceives Aβ fibril or plaque stiffness, which stimulates a protective response to Aβ pathology and mitigates the progression of AD. Thus, microglia Piezo1 may be a prospective therapeutic target for AD treatment.

### 4.4 Piezo1‐mediated mechanotransduction in brain tumors

Malignant tumors are characterized by a complete destruction of the normal tissue and corresponding alterations of cellular mechanics, including ECM remodeling and sclerosis (Miroshnikova et al., 2016). Unlike tumors such as liver, lung, or breast cancer that present with increased stiffness, brain tumors have a marked heterogeneity in their mechanical properties, which is related to the origin and location of the tumor. Hughes et al. (2015) used magnetic resonance elastography to detect tumor stiffness in meningioma patients and found gradual changes in the stiffness of meningiomas, which was heterogeneous in different regions. In addition, human gliomas exhibit increased tissue stiffness as they progress from lower‐grade glioma (LGG) (50–1400 Pa) to glioblastoma (GBM) (70–13,500 Pa) (Miroshnikova et al., 2016). Due to this change, tumor cells may experience high local stiffness in gliomas (Pogoda & Janmey, 2018).

Indeed, increased stiffness is a physical hallmark of most solid tumors, which further activates signaling pathways associated with tumor cell proliferation, invasion, drug resistance, metabolism, and growth promotion, contributing to tumor progression (Barnes et al., 2017; Northey et al., 2017; Tung et al., 2015). Glioma, the most common brain tumor, exhibits a progressive increase in tissue stiffness which may contribute to its rapid progression and severe functional deterioration in patients (Chen et al., 2018; Li et al., 2015). Tumor cells generally sense the abnormal physical microenvironment through mechanoreceptors and translate mechanical signals into intracellular signals (Li et al., 2015). A recent study reported that *Drosophila* Piezo (dPiezo) is required for glioma sclerosis and proliferation (Chen et al., 2018). Both bioinformatics and clinical data showed that Piezo1 expression was upregulated in all histological subtypes of glioma, from LGG to GBM, and that its expression was positively correlated with malignancy (Chen et al., 2018; Zhou et al., 2020). Less malignant isocitrate dehydrogenase‐mutant gliomas typically show lower expression of Piezo1, which is associated with hypermethylation upstream of the Piezo1 promoter (Chen et al., 2018). Bioinformatics analysis suggested that genes associated with ECM recombination, cell adhesion, integrin binding, angiogenesis, cell migration, and proliferation are closely related to Piezo1 (Chen et al., 2018; Zhou et al., 2020). Integrins are heterodimeric transmembrane glycoprotein receptors with α and β subunits that act as mechanical stress transducers (Cooper & Giancotti, 2019). Integrin‐dependent kinases (FAKs) are a key kinases in the integrin pathway. The effect of integrins on pathways, such as FAK/Ras/MAPK, FAK/PI3K, and FAK/STAT, are known to be crucial for regulating tumor cell proliferation, gene transduction, and apoptosis (Cooper & Giancotti, 2019). In *Drosophila* glioma models ranging from LGG to HGG, dPiezo interacts with the integrin‐FAK signaling pathway and participates in regulating ECM remodeling, which in turn further increases tissue stiffness and tumor cell proliferation (Chen et al., 2018). This is a positive feedback process, and activation of Piezo1 may be central to exacerbating tissue sclerosis and promoting tumor progression (Figure 5).

Piezo1 plays a dual role in regulating cell division and death in a steady state to maintain a stable number of cells in the tissue. Activation of Piezo1 in overcrowded epithelial tissues promotes live cell extrusion and death, which is a potential mechanism for the inhibition of tumor cell proliferation (Eisenhoffer et al., 2012; Gudipaty et al., 2017). However, when cells are stretched or at low density, Piezo1 activation promotes cell division (Gudipaty et al., 2017). This Piezo1‐dependent cell proliferation effect is dependent on the suppression of the retinoblastoma gene (Aykut et al., 2020). Upon tumorigenesis, this function of Piezo1 is lost and Piezo1 activation appears to exert antitumor contact inhibition to increase tissue density and unidirectionally promote malignant progression (Chen et al., 2018). Brain tumors are also composed of different cell types, which is one of the reasons for the mechanical heterogeneity of different brain tumors. For example, neuroblastomas, ventricular meningiomas, medulloblastomas (MBs), and malignant gliomas mainly originate from OPCs, neuroblasts, NSCs, and astrocytes, and rarely from differentiated neurons (Liu & Zong, 2012). It is therefore of scientific and clinical significance to determine the effect of mechanical signaling on various brain tumor cells and to identify the mechanism of mechanical transduction.

Piezo1 function is evolutionarily conserved across metazoans, so both human and mouse Piezo1 rescue endogenous piezo1 knockout‐induced glioma growth inhibition in *Drosophila* (Chen et al., 2018). In addition to brain tumors, many other solid tumors overexpress Piezo1, and Piezo1‐mediated mechanotransduction is a general mechanism through which tumors respond to mechanical signals in abnormal tissue (Karska et al., 2023). Loss of Piezo1 in bone marrow cells has been shown to prevent cancer (Aykut et al., 2020). Therefore, specific pharmacological inhibition of Piezo1 may offer hope for the treatment of cancer, including brain tumors.

### 4.5 The role of Piezo1‐mediated mechanotransduction in brain injury

Traumatic brain injury (TBI) denotes a non‐degenerative and non‐congenital injury of the brain caused by external mechanical forces that can result in permanent or temporary impairment of cognitive, physical, and psychosocial functions (Hellawell et al., 1999). Following a traumatic injury (e.g., impact), glial scarring occurs and brain stiffness is altered (Moeendarbary et al., 2017). Studies have shown that the elastic modulus of cortical tissue in healthy rats ranges from 50 to 5000 Pa and is decreased after brain injury (Moeendarbary et al., 2017). Molecular markers of glial scarring were strongly upregulated 9 days after brain injury, and AFM showed that inconsistent with scarring in other parts of the body, glial scar tissue was softer than surrounding areas (Moeendarbary et al., 2017). As mentioned earlier, axonal growth is facilitated in a harder environment, and conversely, lower stiffness of brain tissue will inhibit axonal regeneration and repair after brain injury (Koser et al., 2016). The signal of tissue softening after brain injury is mediated by Piezo1, and knocking down Piezo1 may promote brain repair after injury (Koser et al., 2016).

Consistent with AD, amyloid plaque deposition and elevated Aβ monomer concentrations were also found in the brains of TBI patients (Johnson et al., 2010; Roberts et al., 1991). Aβ40 and Aβ42 are known to be the major aggregating monomers of Aβ in vivo. Physiological levels of monomeric amyloid Aβ40 and Aβ42 improve synaptic plasticity and promote neuronal survival (Giuffrida et al., 2009; Parihar & Brewer, 2010). Increased Aβ monomer concentrations after brain injury inhibit the response of Piezo1 to mechanical signals and eliminate the undesirable effect of softened tissue on nerve regeneration (Maneshi et al., 2018). This suggests that the neuroprotective effect of Aβ monomer after brain injury may be related to the inhibition of Piezo1.

**FIGURE 6:**
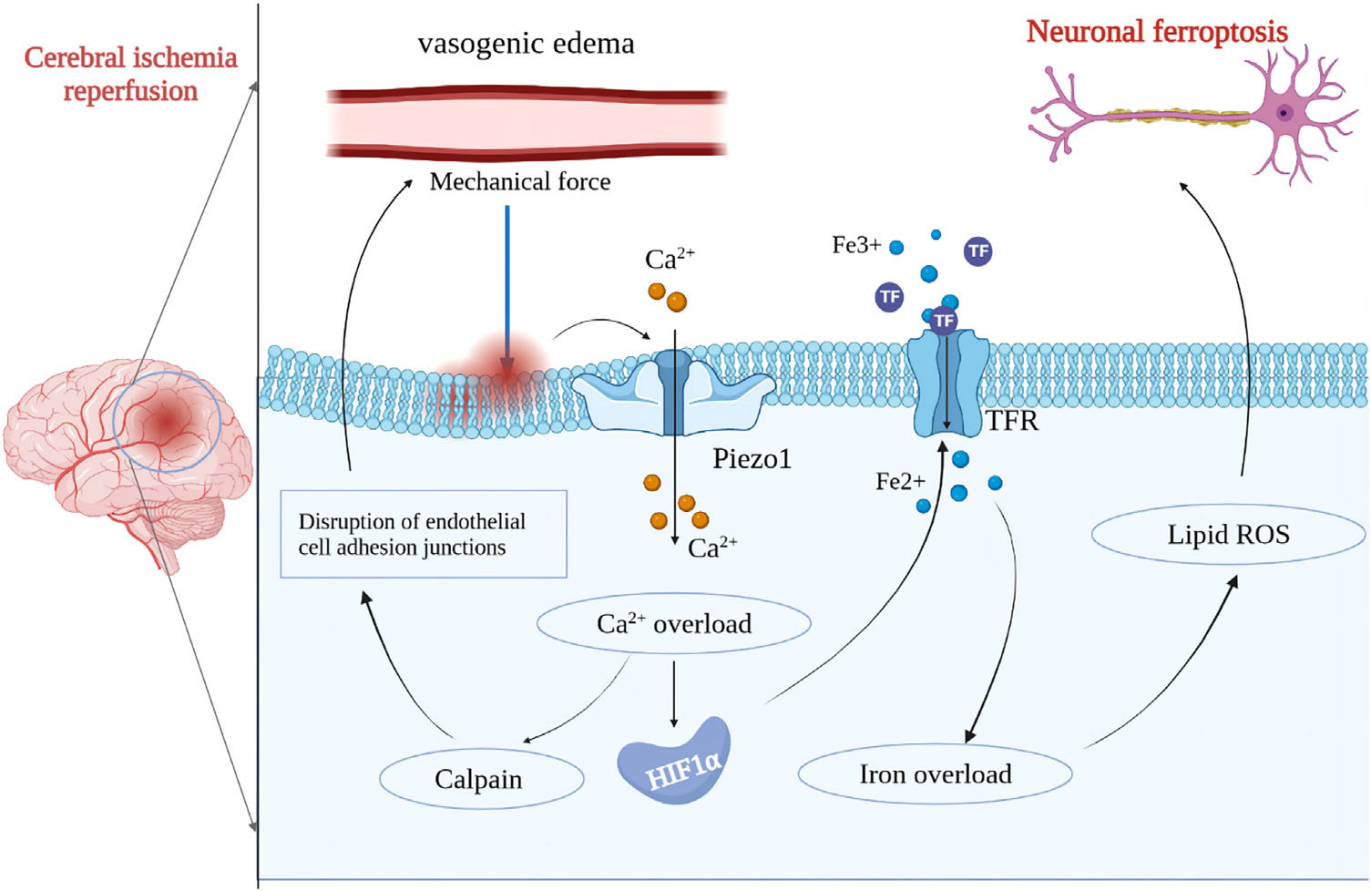
**Illustration of the hypothesis that Piezo1 is involved in brain injury after ischemia–reperfusion through regulation of ferroptosis**. Cerebral ischemia induces vasogenic brain edema, which in turn increases vascular pressure and possibly activates the Piezo1 voltage channel on the neuronal surface. Piezo1 channels convert external mechanical signals into intracellular Ca^2+^ signals and promote hypoxia‐inducible factor‐1α (HIF1α) expression by increasing Ca^2+^ influx, which in turn promotes iron uptake by transferrin receptor (TFR), leading to intracellular iron overload and neuronal ferroptosis (Guo et al., 2021). In addition, Piezo1 activation‐mediated Ca^2+^ overload activates the calpain pathway further exacerbating the disruption of endothelial adhesive junctions, which in turn facilitates vasogenic edema.

Recent studies have shown that Piezo1 is also involved in post‐ischemia–reperfusion injury in the brain (Wang et al., 2019). A rat model of middle cerebral artery occlusion showed increased cortical Piezo1 expression, whereas in an oxygen‐glucose deprivation/reoxygenation (OGD/R) model, it was shown that Piezo1 regulates apoptosis after neuronal OGD/R injury via the Ca^2+^/calpain signaling pathway (Wang et al., 2019). Recently, Wang et al. proposed that Piezo1 channel proteins may be involved in regulating brain injury after ischemia–reperfusion by participating in the regulation of ferroptosis (Guo et al., 2021). Cerebral vascular‐derived edema or thrombus induced after cerebral ischemia–reperfusion exerts mechanical forces on the surrounding tissue (Liu et al., 2020; Wang et al., 2019). Neurons sense changes in external mechanical forces through mechanosensitive Piezo1 channels on the membrane surface, which in turn mediate Ca^2+^ signaling. Studies in myeloid cells have shown that Piezo1‐mediated Ca^2+^ overload promotes the accumulation of hypoxia‐inducible factor‐1α (HIF1α) (Solis et al., 2019), which in turn promotes iron uptake by transferrin receptor (TFR), leading to intracellular iron overload and neuronal ferroptosis (Figure 6) (Guo et al., 2021). Ferroptosis is involved in regulating the expression of essential proteins in cerebral ischemia, and it is necessary to further verify whether Piezo1 can indeed regulate ferroptosis‐mediated cerebral ischemic injury through the HIF1α‐TFR pathway.

Piezo1‐mediated mechanotransduction is also involved in the regulation of cerebral microvascular hydrostatic pressure and vasogenic edema after brain injury. Previous studies have shown that Piezo1 mediates intracellular Ca^2+^ influx in pulmonary capillary endothelial cells, further activating Ca^2+^‐dependent calpain signaling (Friedrich et al., 2019). Increased calpain activation promotes vasogenic edema by promoting disruption of endothelial adhesive junctions and increases vascular permeability promoting extravasation of protein‐rich fluid, leading to vascular permeability (Friedrich et al., 2019). In addition, studies have reported a key role for Piezo1 in peripheral arterial blood pressure regulation by activating NOS and increasing the release of the vasodilator NO (Wang et al., 2016). A recent study showed that Piezo1 localized in the cerebral cortex and retinal capillaries can also act as a mechanosensor in CNS capillaries sensing external signals released by neurons, inducing transient Ca^2+^ currents in endothelial cells, and playing a key role in the regulation of cerebral blood flow (Harraz et al., 2022). Therefore, inhibition of Piezo1 channel activation may alleviate vasogenic edema and increased intracranial pressure after brain injury.

## 5 MODULATION OF PIEZO1 VOLTAGE CHANNELS BY DRUGS

Ruthenium rDIed, Gd^3+^, or the spider peptide toxin GsMTx4 inhibit Piezo1 channels but are not Piezo1‐specific and also act on a variety of other ion channels with mechanosensitive properties (Chen et al., 2018). GsMTx4 is a spider venom peptide that inhibits cationic mechanosensitive channels, including Piezo1 (Gnanasambandam et al., 2017). The inhibition of Piezo1 by GsMTx4 does not depend on direct blockage of the central ion pore, but rather on altering the tension distribution near the Piezo1 channel by inserting itself into the cell membrane (Gnanasambandam et al., 2017). As a result, the efficiency of signal transfer from the lipid bilayer to the Piezo1 channel is reduced by GsMTx4. By contrast, Yoda1 and Jedi1/2 are chemical agonists that act directly on Piezo1 channels to exert their activating effects. Yoda1 was first identified to directly activate Piezo1 protein in the absence of a mechanical force without other cellular components (Syeda et al., 2015). Jedi1/2 activates Piezo1 through the outer upstream propeller blades (Wang et al., 2018), whereas Yoda1 acts on the downstream beam, resulting in currents with a faster burst and decay than Yoda1‐mediated currents. Recent studies have shown that Piezo1 activity is also regulated by dietary fatty acids, with saturated margaric acid inhibiting Piezo activation by increasing membrane bending, and PUFAs in fish oil inducing Piezo1 channel inactivation by reducing membrane stiffness (Romero et al., 2019).

## 6 CONCLUSIONS AND FUTURE DIRECTIONS

Since the identification of the Piezo protein family, research on the mechanosensitive Piezo1 channel has proliferated and achieved significant breakthroughs in recent years. In particular, the role of Piezo1 in the regulation of brain development and CNS diseases has received widespread attention. This may help identify potential therapeutic targets for brain diseases. Accumulating evidence suggests that the brain is a mechanically sensitive organ and that Piezo1‐mediated mechanotransduction has a wide range of effects on its structure and function (Chi et al., 2022; Harraz et al., 2022; Procès et al., 2022), for instance during neuronal axon formation (Song et al., 2019) and neurogenesis (Chi et al., 2022). In this review, we summarized the role of Piezo1 in various cell types of the CNS and described the pioneering research of mechanical properties in the regulation of brain pathophysiology.

The discovery of Piezo1 has led to novel approaches for treating CNS disorders. For example, inhibition of Piezo1‐mediated mechanical damage signaling during aging can rescue the stiff niche‐mediated decline of neurogenesis and promote the functional recovery of NSCs (Segel et al., 2019). In gliomas, Piezo1‐mediated mechanotransduction forms a positive feedback loop of tissue sclerosis and tumor progression (or invasion), and inhibition of Piezo1 or its downstream effectors can effectively mitigate glioma progression (Chen et al., 2018). Suppression of Piezo‐mediated dysregulation of transient Ca^2+^ currents in demyelinating diseases may promote axonal regeneration and myelin formation, as well as limit excessive T cell‐mediated autoimmune responses (Jairaman et al., 2021; Velasco‐Estevez et al., 2020). The role of Piezo1 in neuroinflammation has recently been demonstrated, with Piezo1 deficiency reducing the levels of LPS‐induced pro‐inflammatory cytokines in microglia (Zhu et al., 2023). However, pharmacological studies on Piezo1 are currently limited, particularly in brain diseases. Given the role of Piezo1 in the CNS, pharmacological studies focusing on Piezo1 will help provide new therapeutic strategies for related diseases. Notably, when using Piezo1 as a therapeutic strategy for brain disorders, the dual role of Piezo1, such as anti‐ versus pro‐inflammatory, needs to be considered (Geng et al., 2021; Zhu et al., 2023).

Another member of the Piezo family, Piezo2, is also expressed in the brain (Coste et al., 2010). Piezo2 mediates tactile perception and is primarily expressed in mechanosensory cells such as primary sensory neurons (Ranade et al., 2014). A recent groundbreaking study published in neuron identified an important role for Piezo2 in MB cells, where it controls the permeability of the blood–tumor barrier (BTB), which prevents the efficient delivery of drugs that would otherwise be effective against brain tumors (Chen et al., 2023). Knockdown of the MB tumor cell‐specific Piezo2 gene disrupted the BTB by promoting the transduction of WNT/β‐catenin signaling (Chen et al., 2023). This suggests that inhibition of Piezo2 channels in MB cells may have a therapeutic effect by disrupting BTB and increasing the uptake of chemotherapeutic agents in brain tumors. There is a synergy between the functions of Piezo1 and Piezo2, as they are epistatically related during myelination in Schwann cells (Acheta et al., 2022). Knockdown of Piezo1 alone promotes Schwann cell‐mediated myelin regeneration, whereas the combined knockdown of Piezo1 and Piezo1 did not affect the regeneration of myelin (Acheta et al., 2022). It can therefore be inferred that Piezo2 is a regulator of myelin formation, whereas Piezo1 is an inhibitor of Piezo2 (Acheta et al., 2022). In addition, Piezo1 and Piezo2 are both robustly expressed in articular chondrocytes, synergistically promoting mechanically mediated Ca^2+^ signaling in chondrocytes and promoting mechanical strain‐mediated cell damage following injury (Lee et al., 2014). However, it is still not clear how the synergistic effects of Piezo1 and Piezo2 play out at the molecular level.

In addition to the Piezo family, other mechanosensitive ion channel proteins, including transient receptor potential (TRP), epithelial sodium channel (ENaC), TREK/TRAAK, and degenerations/acid‐sensitive channels, are also expressed in the CNS (Javier‐Torrent et al., 2021; Yang et al., 2022). In general, different mechanoreceptors respond to specific mechanical stimuli, such as ENaC in endothelial cells being activated in response to shear force, tension, and hydrostatic pressure to regulate blood pressure (Yang et al., 2022). By contrast, TRPV1 senses pain and temperature stimuli (Gavva et al., 2007). Piezo1 responds to various forms of mechanical stimuli, such as stretch, shear, and substrate stiffness. The TRP channel protein family is well‐studied in the CNS. TRVP4 is also an ion channel sensitive to matrix stiffness, activated by stimulation at cell‐matrix contacts, but insensitive to mechanical indentation and membrane stretching (Servin‐Vences et al., 2017; Sharma et al., 2020). Thus, TRP channels are more likely to be activated as mechanical downstream components, acting as amplifiers of mechanosensory signaling cascades. There is evidence that TRPV4 acts downstream of Piezo1 following its activation (Swain & Liddle, 2021; Swain et al., 2020). Activation of Piezo1 in pancreatic alveolar cells further activates TRPV4 channels and leads to sustained elevation of intracellular Ca^2+^ levels, which in turn leads to organelle dysfunction (Swain et al., 2020). In endothelial cells, activation of TRPV4 channels is dependent on shear forces and Piezo1 activation to generate sustained Ca^2+^ flow, whereas Piezo1 channel‐mediated tight junction disruption is dependent on TRPV4 activation (Swain & Liddle, 2021). In odontoblasts and neurons, Piezo1 and TRPV1 act synergistically to regulate dentinal pain (Ohyama et al., 2022). TRVP1‐mediated Ca^2+^ influx in DRG neurons inhibits Piezo1 and Piezo2 channels by depleting membrane phosphoinositides (Borbiro et al., 2015). In addition, studies have found an important role of the TRPV family in the regulation of neurodegenerative diseases (Montell, 2001). Further studies are needed to determine whether Piezo1 acts in synergy with TRPV‐mediated mechanical signaling to promote brain disease.

To date, studies of Piezo1‐mediated mechanotransduction in the CNS to regulate brain physiopathology are still in their infancy, and there are still many elusive aspects of the role of voltage channels in the brain. First, the vast majority of current work on Piezo1 channels in the CNS relied on the phenotypic analysis of knockdown systems or agonists and inhibitors (Malko et al., 2023; Velasco‐Estevez et al., 2020; Zhu et al., 2023). Exactly how Piezo1 uses its mechanosensitivity to regulate pathophysiological functions in the brain needs to be studied in more depth. Second, the site and specific mechanism of Piezo1 and Aβ action are still unclear. In addition, further studies are needed to investigate the role of Piezo1 channel‐mediated mechanical signaling in other neurodegenerative diseases, including Parkinson's disease, Huntington's chorea, and amyotrophic lateral sclerosis. Third, the physicochemical properties of matrix stiffness significantly contribute to neuronal cell fate during mechanical stimulation (Procès et al., 2022). Changes in the mechanical environment of the brain can lead to age‐related cognitive decline and neurodegenerative lesions (Chi et al., 2022). Further research is required to understand how changes of matrix composition and mechanical properties relate to neuronal pathophysiology and cognition. Finally, considering the significant upregulation of neuronal Piezo1 expression in the DG and CA1 regions of the hippocampus of aging AD rats (Velasco‐Estevez et al., 2018), further investigation of the relationship between neuronal Piezo1 expression and cognitive function is warranted. These questions will hopefully inspire new research on the mechanism of Piezo1‐mediated mechanotransduction in the CNS and provide new directions for the treatment of CNS diseases.

The brain cannot perform its functions without a stable mechanical environment (Javier‐Torrent et al., 2021). The widely expressed mechanosensitive Piezo1 channel is an ideal mechanosurveillance system for precise monitoring of subtle changes in the mechanical environment of the brain. The Piezo1 mechanoreceptor recognizes changes in environmental stress and translates mechanical changes into intracellular electrochemical signals that control key neurobiological processes. Piezo1 loss of function or overactivation plays a crucial role in the pathogenesis of many common brain diseases. Pharmaceutical drugs and other therapeutic approaches targeting Piezo1 will show considerable promise. However, the expression and function of Piezo1 in various types of brain cells need to be further clarified. In particular, the role of Piezo1 in different physiological and pathological states of the brain will be the focus of future research.

## AUTHOR CONTRIBUTIONS

Qingcui Zheng and Hailin Liu performed the literature search and manuscript writing. Wen Yu, Yao Dong, Lanqian Zhou, and Wenze Deng edited and revised the manuscript. Fuzhou Hua guided and revised this manuscript. All authors have read and agreed to the published version of the manuscript.

## CONFLICT OF INTEREST STATEMENT

The authors declare that the research was conducted in the absence of any commercial or financial relationships that could be construed as a potential conflict of interest.

### PEER REVIEW

The peer review history for this article is available at https://publons.com/publon/10.1002/brb3.3136.

## Data Availability

N/A
